# Parallel evolution of alternate morphotypes of *Chryseobacterium gleum* during experimental evolution with *Caenorhabditis elegans*

**DOI:** 10.1093/femsec/fiae039

**Published:** 2024-03-28

**Authors:** Marissa Duckett, Megan N Taylor, Claire Bowman, Nic M Vega

**Affiliations:** Department of Biology, Emory University, 1510 Clifton Road NE #2006, Atlanta, GA 30322, United States; Department of Biology, Emory University, 1510 Clifton Road NE #2006, Atlanta, GA 30322, United States; Department of Biology, Emory University, 1510 Clifton Road NE #2006, Atlanta, GA 30322, United States; Department of Biology, Emory University, 1510 Clifton Road NE #2006, Atlanta, GA 30322, United States; Department of Physics, Emory University, 400 Dowman Dr, Atlanta, GA 30322, United States

**Keywords:** *Chryseobacterium gleum*, evolution, experimental, microbial communities, morphotypes

## Abstract

Microbial evolution within polymicrobial communities is a complex process. Here, we report within-species diversification within multispecies microbial communities during experimental evolution with the nematode *Caenorhabditis elegans*. We describe morphological diversity in the target species *Chryseobacterium gleum*, which developed a novel colony morphotype in a small number of replicate communities. Alternate morphotypes coexisted with original morphotypes in communities, as well as in single-species experiments using evolved isolates. We found that the original and alternate morphotypes differed in motility and in spatial expansion in the presence of *C. elegans*. This study provides insight into the emergence and maintenance of intraspecies diversity in the context of microbial communities.

## Teaser

Bacterial diversity evolves repeatedly within microbial communities, and intraspecies diversity is not obvious from community-level diversity.

## Introduction

How microbes adapt within networks of biotic interactions is an ongoing question. During experimental evolution, microbial communities in a given environment tend to follow similar trends in composition over time, where the same or similar taxa are present across communities (Morella et al. 2019, Estrela et al. 2021, Taylor et al. 2022). Across experiments, conditions, communities, and contexts, the community-level trends observed during evolution are similar: a rapid initial shift in community composition is followed by more gradual changes, generating a characteristic fast–slow pattern in compositional divergence from the initial community (Goldford et al. 2018, Estrela et al. 2021). This suggests that evolution of microbial communities follows rules, such that similar selective pressures lead to community-level structural similarities (Meroz et al. 2021).

However, it does not follow that individual member taxa must evolve similar traits. The genetic and phenotypic traits of specific taxa within communities often diverge and diversify (Castledine et al. 2020). One example is within-host evolution in host-associated microbial communities, where a given microbe of interest will evolve over time *in situ*. In some cases, selective sweeps result in different communities harboring a different dominant off-shoot of the same common ancestor (Didelot et al. 2016); the result is observed as diversity between hosts (Didelot et al. 2016) or across sites within the same host (Conwill et al. 2022). Alternately, variants from a common ancestor can coexist, producing within-population diversity (Rainey et al. 2000, Chu et al. 2021, Turner et al. 2023). A multispecies community context can promote within-species diversity (Chu et al. 2021), but as diversity can emerge from homogeneous populations (Cano et al. 2003, Hoffman et al. 2006, Blount et al. 2008, Pestrak et al. 2018, Chen et al. 2019, Obeng et al. 2023), a multispecies community is not required. The conditions for adaptive diversification are not yet clear.

Adaptation is driven by pressures in the abiotic and biotic environment, including interactions with eukaryotes. Here we use *Caenorhabditis elegans*, an established model system for microbial coevolution experiments (Meneely et al. 2019, Ford and King 2020, Gibson et al. 2020), as a host and predator during microbial community evolution with the worm. As in previous work, we use combinatorial, defined starting communities with a tractable number of members (Taylor et al. 2022), allowing us to follow evolution within member taxa.

In this study, we report parallel evolution of within-species diversity in bacteria from *C. elegans*-associated communities. We focus on characterization of a morphological variant in the Gram-negative opportunistic pathogen *Chryseobacterium gleum*. The variant emerged at similar times in different communities and was maintained through the end of the experiment. The alternate morph differed in motility when compared to the original morphotype, as well as in population expansion on solid media in the presence of *C. elegans*. Our results provide insight into the selective pressures that can produce and maintain intraspecies variation during community-based microbial evolution.

## Materials and methods

### Strains and culture conditions

Bacterial strains in Table 1 were obtained from the USDA Agricultural Research Service (ARS) Culture Collection (NRRL) or from the American Type Culture Collection (ATCC). Strains showed characteristic morphologies on salt-free nutrient agar (NA; 3 g yeast extract, 5 g peptone, and 15 g/l agar), which was used for identification of colonies (Taylor et al. 2022). Combinatorial communities were set up with mixtures of seven strains as shown in Table 2.

**Table 1.** Bacterial strains used in this study.

ID	Abbreviation	Taxonomic Name	Source
B-2879	AA	*Arthrobacter aurescens*	NRRL
B-24236	MO	*Microbacterium oxydans*	NRRL
B-16025	RE16	*Rhodococcus erythropolis*	NRRL
B-1574	RE15D, RE15M	*Rhodococcus erythropolis* (dry, mucoid)	NRRL
B-1876	BS	*Bacillus sp*.	NRRL
ATCC 49188	OA	*Ochrobactrum anthropi*	ATCC
B-14798	CG	*Chryseobacterium gleum*	NRRL
B-14848	CI	*Chryseobacterium indologenes*	NRRL
B-14902	ST	*Sphingobacterium thalpopium*	NRRL
B-23392	SS	*Sphingobacterium spiritovorum*	NRRL

**Table 2.** Combinatorial community composition. Rows are specific composition of 12 combinatorial communities, A–L. AA, *Arthrobacter aurescens*; MO, *Microbacterium oxydans*; RE15M/D, *Rhodococcus erythropolis* B-1574 mucoid (M) or dry (D); RE16, *R. erythropolis* B-16025; OA, *Ochrobactrum anthropi*; CG, *Chryseobacterium gleum*; CI, *C. indologenes*; SS, *Sphingobacterium spiritovorum*; and ST, *S. thalpopium*.

Multispecies combinations							
**A**	AA	MO	RE15D	BS	OA	CG	SS
**B**	AA	MO	RE15D	BS	OA	CG	ST
**C**	AA	MO	RE15D	BS	OA	CI	SS
**D**	AA	MO	RE15D	BS	OA	CI	ST
**E**	AA	MO	RE15M	BS	OA	CG	SS
**F**	AA	MO	RE15M	BS	OA	CG	ST
**G**	AA	MO	RE15M	BS	OA	CI	SS
**H**	AA	MO	RE15M	BS	OA	CI	ST
**I**	AA	MO	RE16	BS	OA	CG	SS
**J**	AA	MO	RE16	BS	OA	CG	ST
**K**	AA	MO	RE16	BS	OA	CI	SS
**L**	AA	MO	RE16	BS	OA	CI	ST

N2 Bristol *C. elegans* were provided by the *Caenorhabditis* Genetics Center, which is funded by NIH Office of Research Infrastructure Programs (P40 OD010440). Stocks of ancestral N2 worms were cultivated on 10 cm NGM agar plates at 25°C with *E. coli* OP50 as a food source according to standard protocols (Stiernagle 2006).

### Community-based experimental evolution

**Figure 1.**
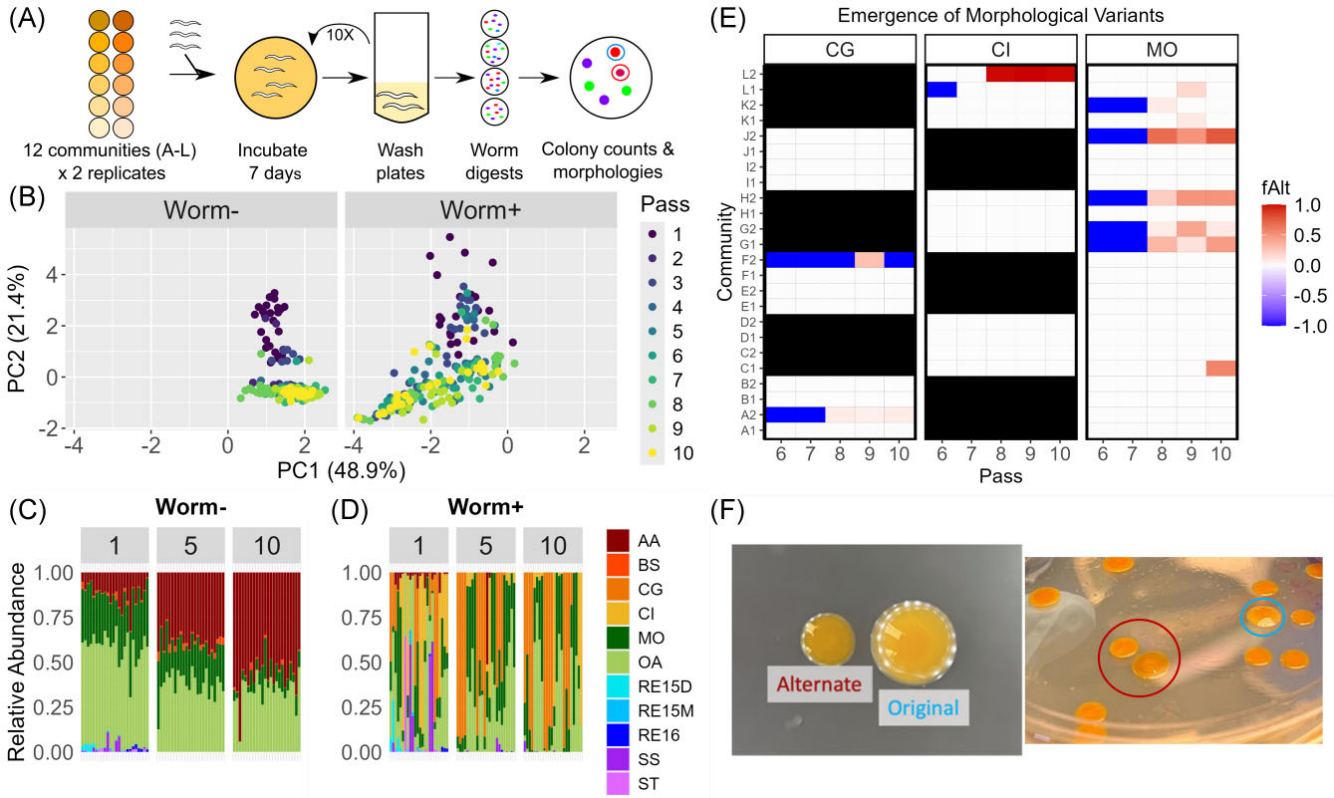
Community-based adaptation produces convergent community composition and within-species diversity. (A) Experimental workflow for community-based evolution. The experimental set up consisted of 12 communities with seven members each, similar at a genus level but variable on a species/isolate level. Each community was initiated on two replicate plates (community A–L, replicates 1 and 2), to which L1 stage N2 *C. elegans* were added. These communities + worm populations were passaged every seven days to fresh NGM agar plates. At the end of each passage, adult worms were batched, physically disrupted to release gut contents, and plated in serial dilution to assess bacterial community composition. (B)–(D) Community composition follows coherent trajectories over time, with compositional differences associated with *C. elegans*. For plates with worms (Worm+), data are from batch digests of adult worms taken from plates at the end of each passage (*n* = 50 adult worms per batch). For plates established without worms, data are for community lawns washed from plates. (B) PCA of community composition data over time (10 passages of experimental evolution) Each point represents one replicate (12 communities, two replicates each, *n* = 24 data points per passage). Data from worm+ and worm− communities were combined for ordination and are plotted separately for clarity. (C) and (D) Relative abundance within microbial communities in passages 1, 5, and 10, (C) without and (D) with *C. elegans*; data are the same as in (B). Communities are shown left to right in order of community ID and replicate number (community A replicate 1, A1; A replicate 2, A2…; total *n* = 24). Bacterial strains are abbreviated: “AA”, *A. aurescens*; “BS”, *B. subtilis*; “CX”, *C. gleum* (CG) or *C. indologenes* (CI); “MO”, *M. oxydans*; “OA”, *O. anthropi*; “RE”, *R. erythropolis*; “SX”, *S. spiritovorum* (SS), or *S. thalpopium* (ST) (Tables 1 and 2). *Chryseobacterium gleum* (CG) is the *Chryseobacterium* isolate in communities A–B, E–F, and I–J (columns 1–4, 9–12, and 17–20). (E) and (F) Morphological diversity in worm+ communities. (E) Emergence of alternate morphs. Passages where alternate morphotypes were observed are colored according to the relative abundance of the alternate morph, as a fraction of that species’ total representation (fAlt). For passages where alternate morphs were noted but not counted, or where alternate morphs were retrieved from glycerol stocks but not from the original worm digest plates, relative abundance is unknown and is assigned a value of −1 (blue) for visualization. Bars (black) indicate communities where the indicated isolate (CI or CG) was not part of the initial community. Community replicates are on the *y*-axis (in reverse order top to bottom, L2→A1). (F) Colony morphologies in *C. gleum*. On the salt-free nutrient agar (NA) used for community plating, the original morph is brightly colored, glossy, domed, smooth, and slightly mucoid. The alternate morph is smaller and flattened, with less opacity and duller coloration. Morphologies are distinctive despite minor variation (right image, from community F1 passage 10). Two colonies with variations on the alternate morphology are circled in the center of the image ; one original morphology colony is circled on the right side of the plate.

Community-based evolution was carried out as in (Taylor et al. 2022), with minor modifications. To observe the outcomes of community-based host-associated evolution in a simple microbial community, we initiated experimental evolution of combinatorial minimal communities of bacteria (see Tables 1 and 2) with and without the nematode *C. elegans* as predator and host (see Fig. 1 for workflow schematic). Starting cultures of bacteria were pregrown individually in 1 ml LB cultures for 48 h at 25°C, transferred to 1.5 ml Eppendorf tubes, and centrifuged 2 min at 9000 × *g* in a tabletop centrifuge (Eppendorf) to pellet. Supernatant was removed and bacteria were resuspended in 1 ml S medium, then diluted to 10^8^ CFU/ml in S medium and combined in equal ratios to form the communities in Table 2. Worm+ plates were initiated with synchronized L1 larvae of WT Bristol N2 *C. elegans* (∼100 L1 larvae per plate). Worms were added to 6 cm NGM agar plates with 50 µl of the indicated community, with UV-killed lawns of *E. coli* OP50 as a starting food source. All communities were established in duplicate.

Every 7 days, plates were scored on a 0–4 scale for number of total worms, number of larvae, and plate coverage by bacterial lawn. After scoring, plates were washed once with 1 ml M9 worm buffer + 0.1% Triton X-100 (M9TX01) to remove the bulk of the worms and the bacterial lawn. The resulting suspension was centrifuged briefly in a small benchtop centrifuge to pellet worms, then washed again with 1 ml M9TX01. The second wash was drawn down to 100 µl and supernatant was discarded; worms were resuspended by flicking, and 10 µl of the worm- and bacteria-containing liquid was transferred to a fresh room-temperature plate of NGM + UV-killed OP50. Adult worms from the remaining sample were used for community analysis; target was 50 individual worms, but if total adult count was insufficient, all adults in the sample were counted and digested. Worms were washed, surface bleached, and mechanically disrupted as batches using a motorized pestle according to standard protocols for this lab (digests in 20 µl M9TX01 in 0.5 ml tubes, Kimble Kontes) (Taylor and Vega 2021).

Samples of live worms and bacterial contents from batch digests were individually frozen on the day of passaging. Samples of the *C. elegans* population were preserved at −80°C according to standard protocols (Stiernagle 2006). Half of the volume from worm batch digests was used for plating on NA to determine community composition; the remaining half was mixed 1:1 with 40% glycerol and frozen at −80°C. Single colony picks were taken from worm digest plates (up to six isolates per species per replicate), grown in 100 µl LB in 96-well plates for 48 h at 25°C, then mixed 1:1 with 40% glycerol and covered with aluminum sealing foil before freezing at −80°C. When additional isolates were needed, the appropriate cryopreserved worm batch digests were streaked out from glycerol on NA plates; single colonies were restreaked on fresh NA plates to confirm morphology, then single colony isolates from homogeneous plates were grown to late log phase in LB or NGM liquid medium and cryopreserved at −80°C.

For community-based evolution in the absence of worms, a slightly modified protocol was used. Plates were initially established with ancestral communities as already described, but on 6 cm NGM plates without UV-killed OP50 as this initial worm food source was not needed. Every 7 days, plates were washed with M9TX01 to remove the entire bacterial lawn, centrifuged for 2 min at 6000 × *g* to pellet, and resuspended in 1 ml S medium by pipetting. The resulting suspension was diluted in a 10-fold series for plating and CFU counting, and 10 µl of a 1:10 dilution of each suspension was aliquoted onto the center of a fresh 6 cm NGM plate to seed communities for the next passage.

### Pairwise bacterial interactions on solid media

Isolates of CG from passage 6 and 10 worm+ communities A1, A2, F2, and F2, as well as the ancestral strain (*n* = 3 isolates per morph per condition), were grown up separately in 1 ml LB cultures for 24 h at 25°C and diluted to a uniform cell density of 10^8^ CFU/ml in S medium. Isolates of the original morph representing a given community + passage were combined. For pairwise competitions, each original morph was mixed with individual alternate morph isolates. Mixed pairs were diluted 1:10 in S medium, then 10 µl of each mixture was transferred to the center of two 6 cm NGM plates (1.5% agar, poured on the day of experiment), and plates were moved to 25°C to grow. At indicated time points (2, 7, and 14 days), lawns were washed off plates (see *Community-based experimental evolution*) and resuspended in 1 ml 1X PBS. These resuspensions were serially diluted 10-fold in 1X PBS (200 µl final volume per well) and plated in 100 µl aliquots on 10 cm NA plates for CFU counting and identification of original and alternate morphs. On day 7, fresh plates were inoculated with 10 µl of the first (1:10) serial dilution of the resuspended plate contents and incubated as described for 7 days for sampling on day 14. To quantify the effects of experimental factors (community, generation, strain ID) on fAlt morph, beta regressions with fAlt as the dependent variable were carried out in R using function *betareg()* from package betareg.

For pairwise interactions in the presence of worms, plates were established as already described, except with the addition ∼10 gravid N2 hermaphrodites to each plate on day 0. Propagation and quantification of *C. gleum* populations was carried out as already described.

To compare bacterial populations within plates, a stainless steel circular clay hole cutter (diameter 0.8 cm) was used to remove agar plugs from the edge and center of each 6 cm plate (one plug at each location per plate sampled). Plugs were deposited directly into 1 ml 1X PBS in 1.5 ml microcentrifuge tubes and vortexed to resuspend bacteria. The resulting suspension was dilution plated onto NA as already described.

### *Chryseobacterium gleum* mortality assays

As we observed host mortality in monocolonization experiments with these isolates, we sought to determine whether *C. gleum* was pathogenic to *C. elegans*, and whether evolved and ancestral isolates differed in pathogenicity. Isolates of *C. gleum* ancestral strain (ANC) as well as isolates obtained from worm+ community plates in passages 6 and 10 were grown separately in 1 ml LB cultures for 48 h at 25°C and diluted to a uniform cell density of 10^8^ CFU/ml. The isolates used in these experiments were the same randomly selected isolates as in the previous section. A volume of 50 µl of each isolate were plated onto separate 6 cm NGM plates in triplicate. Plates were grown up at 25°C for 48 h to develop a lawn. Using a BioSorter (250FOCA, Union Biometrica), 50 sucrose washed adult N2 worms were plated onto the resulting lawns. Plates were incubated at 25°C, and worms were scored every 24 h for 4 days. Worms that were not moving independently and did not respond to prodding with a worm pick were scored as dead.

### Growth curves

Bacteria isolated from evolved communities and ancestral isolates for each strain were grown up separately in 1 ml LB cultures for 24 h at 25°C, then diluted in liquid NGM to a uniform cell density of 10^8^ CFU/ml. Each strain was then further diluted 1:100 to a final cell density of 10^6^ CFU/ml in 150 µl liquid NGM in a clear polypropylene 96-well plate (three to four replicates per strain, each from a separate single colony isolate). The plate was covered with BreatheEasy Sealing Membrane and incubated on a BioTek Synergy HTX plate reader using a 24-h kinetic protocol reading OD600 every 10 min at 25°C. Growth curve data were analyzed in R using the function *SummarizeGrowthByPlate*() in *growthcurver* (Sprouffske and Wagner 2016) to infer carrying capacity of the logistic growth model from the OD600 over time data from each individual well; maximum growth rates were inferred for each well using *many.splines.fit()* from package *growthrates* (Hall et al. 2014). Parameter values were calculated for all replicates for each individual strain, and summary statistics (mean, median, and variance) were calculated across experiments for each replicate.

### Surface motility

Surface motility was assayed using a canonical drop culture assay on 6 cm NGM agar plates with 0.35%, 0.5%, or 1.5% agar. Plates, 6 cm, were filled with precisely 8 ml of agar on the day of experiment and allowed to solidify lid-off for 30 min in a laminar flow hood to help ensure uniform surface moisture across plates (Tremblay and Déziel 2008). Bacterial cultures were grown overnight from glycerol stocks at 25°C with shaking at 300 RPM in 100 µl of LB in individual wells of a 96-well plate, covered with a BreatheEasy Sealing Membrane (USA Scientific). To acclimate cultures to the growth medium, cultures were diluted 1:1000 into 100 µl liquid NGM the morning of the experiment and allowed to regrow under the same growth conditions for 4 h. To inoculate motility plates, 1 µl drops of NGM culture were carefully placed in the center of each plate, avoiding splatter. Plates were incubated in stacks of not more than three plates at 25°C in the dark, with a tray of water providing ambient humidity. 1.5% and 0.5% plates grew for 7 days. 0.35% plates grew for 2–3 days due to the swarms reaching the edge of the agar plate. Swarm diameter was measured with a ruler at 2–3 h intervals except overnight.

In assays to determine the effects of nutrient concentration on motility, the same protocol was followed, with the exception that 0.35% NGM agar was made with peptone at 0.2X (0.5 g/l), 1X (2.5 g/l), or 4X (10 g/l) of the standard formulation. All other components were kept constant according to the standard formulation for NGM agar. Acclimatization was done in 0.2X NGM. Where indicated, 10-fold dilutions of NGM cultures were made immediately prior to inoculation of motility plates.

Data were fitted to linear models. For plates established with a constant inoculum, the full model (Diameter ∼ log(Time) + Peptone + Morph + Community) was compared using likelihood ratio testing (LRT) against nested models dropping each independent predictor individually to determine which predictors were significant. For plates initiated with a range of initial inoculum densities, the full linear regression model was instead Diameter ∼ log(Time) + Peptone + Morph + Dilution; only community-A isolates appeared in this data set. LME models were fitted to data for original and alternate morphs separately to determine whether dilution of the initial inoculum had a significant effect on slope, intercept, or both in the linear model. Linear regressions were carried out in R using the function *lm()* from package *stats* in base R, and LME fits were carried out using the function *lmer()* from package *lme4*.

### DNA extraction and genome sequencing

Cultures for genome extraction were grown individually overnight in 1 ml cultures of LB (25°C, shaking at 300 RPM). Samples were centrifuged at 13 000 RPM for 2 min to pellet cells. Cells were washed with 1 ml 1X PBS three times, resuspended in 1 ml PBS, and separated into 5 PCR tubes (200 µl each). To improve lysis efficiency, a freeze–boil protocol was used (−80°C freezer for 10 min, then 100°C thermocycler for 10 min; repeat). DNA extraction was carried out using the Promega Wizard DNA Extraction Kit, with addition of 10 µl proteinase K (20 mg/ml, MolBio or Invitrogen) and 50 µl lysozyme (20 mg/ml, MolBio) to the lysis step (incubated 30 min at 37°C in bead bath to allow lysis to proceed) (Shehadul Islam et al. 2017). Samples were rehydrated with 50 µl rehydration solution for 1h at 65°C. DNA concentration of each sample was measured with both Qubit (Invitrogen Qubit High Sensitivity Assay) and Nanodrop (BioTek Synergy HTX plate reader, Take3 plate). Samples above 20 ng/µl were sent to Microbial Genome Sequencing Center (SeqCenter) for 200Mbp Illumina sequencing with NovaSeq 6000.

Quality control and assembly were performed in Bactopia (Petit and Read 2020) using default parameters, with reference to a species-specific dataset (Ariba reference datasets, RefSeq mash sets, and GenBank sourmash) including full genome assemblies of up to 1000 genomes identified as *C. gleum*. Variant calling was performed against the complete reference genome for each strain sequenced.

Mauve was used to identify SNPs from multiple genome alignment of the evolved isolates and the ancestral genome. The alignment with the reference genome was used for identification of SNP-containing loci when possible. When no homolog was available in the reference genome, or when homologs were not annotated with gene names or specific functions, nearest homologs were identified via nucleotide megaBLAST (high homology) or BLASTn (somewhat dissimilar sequences) against *C. gleum* and *C. cucumeris*.

## Results

As in previous work (Taylor et al. 2022), 12 combinatorial communities were constructed from combinations of 11 member strains. All communities initially contained seven members, representing the same set of genera but with different representatives at the species or strain level (Tables 1 and 2). This design allowed us to compare outcomes for each strain across at least four to six compositionally similar communities.

Experiments were initiated by inoculating communities in technical duplicate on NGM agar plates with wild type (WT) N2 *C. elegans* (Fig. 1A). Communities were passaged to fresh plates every 7 days, at which time a subsample of adult worms from each plate was disrupted to release intestinal contents (Taylor et al. 2022) to quantify community composition (see the section “Materials and methods”). Single-species evolution, where each of the eleven strains was passaged individually on plates with worms, was carried out in a previous run of this experiment (Taylor et al. 2022) and was not repeated here.

As previously observed (Taylor et al. 2022) experimental passaging of combinatorial bacterial communities with *C. elegans* resulted in coherent trajectories of community composition, which were largely robust to species-level variation in the starting communities (Fig. 1B–D). These trajectories were characterized by rapid loss of community richness, resulting in communities comprised almost entirely of *C. gleum/indologenes* (CX), *Microbacterium oxydans* (MO), and *Ochrobactrum anthropi* (OA) within the first five passages. Composition converged to one of two states; communities initiated with *C. gleum* came to be dominated by that species, whereas communities initiated with *C. indologenes* were dominated by *M. oxydans* and *O. anthropi* (Fig. 1D). While compositional convergence was also observed in communities passaged without *C. elegans* (Fig. 1C), community composition was very different without worms, indicating that *C. elegans* played an important role in shaping these communities.

We observed diversification within member taxa in worm-associated communities, in the form of morphological colony variants of *Chrysebacterium* and *M. oxydans* (Fig. 1E and F; Figure S1, Supporting Information). No communities produced variants of both *Chryseobacterium* and *M. oxydans*. Not all communities produced variants; in most cases, only one of a given pair of replicate communities did so. With some exceptions, variants emerged nearly in synchrony across communities around passage 6, and morphotypes frequently coexisted to the end of the experiment. We did not observe colony variants in communities passaged without worms (Fig. 1) or during passaging of single bacterial strains on plates with worms but without the community (Taylor et al. 2022). Although there was some variation within each morphotype (Fig. 1F; Figure S1, Supporting Information), alternate morphs from different communities were visually similar, leading us to classify colonies into “original” (same morphology as ancestor) and “alternate” morphs. Timing of *Chryseobacterium* variant emergence corresponded roughly with time-series minimums of compositional diversity (Figure S2, Supporting Information).

To better understand the selective pressures underlying diversification in these populations, we chose to focus on characterization of within-species diversity in *C. gleum* (CG). Morphological diversity in *Chryseobacterium* emerged only twice, but from two different starting communities, allowing us to compare isolates across these different initial conditions. *C. gleum* alternate morphs were observed in community A replicate 2 (A2) and community F replicate 2 (F2); the remaining replicate of both communities A and F (A1, F1) generated only colonies of the original morphotype. Taxonomy of evolved isolates was confirmed via 16S sequencing. Isolates from both morphotypes retained full pathogenicity toward the worm (Figure S3, Supporting Information), consistent with our prior experience with these *Chryseobacteria* (Taylor et al. 2022) and with other work in this clade (Page et al. 2019).

### Differences between morphotypes

#### Logistic growth

We first sought to explain intraspecies diversity in *C. gleum* from an ecological standpoint, to determine (1) how intraspecies diversity is maintained in these populations and (2) whether the emergence of diversity in one replicate of each diversified community has an ecological explanation, or simply reflects the low frequency at which new variants arise. We began with the simplest hypothesis, where intraspecies diversity is maintained or lost due to interactions with conspecifics.

Bacterial growth parameters (maximum growth rate and saturation density) are often used as markers of relative fitness (Concepción-Acevedo et al. 2015, Momeni et al. 2017, Saavedra et al. 2017). We, therefore measured growth parameters for original and alternate morphotype isolates of *C. gleum*, from passages 6 (early emergence) and 10 (end of experiment) and communities A and F. Liquid NGM medium and growth at 25°C were used to approximate the conditions in the original experiment. Individual isolates were grown in separate wells for 24 h in a plate reader to record absorbance (OD_600_) over time, and cultures were dilution plated at the end of the run to determine colony forming units (CFU/ml).

Overall, original and alternate morphotypes showed minimal differences in growth (Figure S4, Supporting Information). Maximum growth rate was lower in alternate morphs than in the ancestral strain; only one set of original morphs (from community F2 pass 10, F2o10) showed this decrease relative to ancestor. CFU measurements from stationary phase cultures indicated increased saturation density in alternate morphs as compared with the ancestor. However, many original morphs (A1o10, A2o10, F1o6, F2o6, and F2o10) also showed higher saturation density than the ancestor, which was not always accompanied by a reduction in maximum growth rate.

These results did not explain the observed morphotype diversity. While a tradeoff between decreased exponential growth rate and increased saturation density in alternate morphs might have allowed coexistence of morphotypes, the improved saturation density and more rapid growth of evolved original morphs should have pushed the balance toward competitive exclusion. Further, these data did not explain the absence of alternate morphotypes in one replicate (A1, F1) of each community.

#### Pairwise competition on solid media

Growth of isolates in liquid media may not be a good representation of performance in mixed cultures in a structured environment. We, therefore next sought to assess intraspecies competitive ability of alternate and original morphs directly, using pairwise competitions on solid media. In these experiments, cultures of alternate and original morphs were grown separately in liquid media, diluted to fixed density, then mixed 1:1 and inoculated into the center of NGM agar plates as was done during experimental evolution. No worms were added during these experiments, to focus only on intraspecies interactions between morphotypes. At indicated time points, plates were washed with buffer to resuspend the entire lawn, and the resulting suspension was plated to determine counts of alternate and original morphologies. Populations were passaged to fresh plates after 7 days as before.

**Figure 2.**
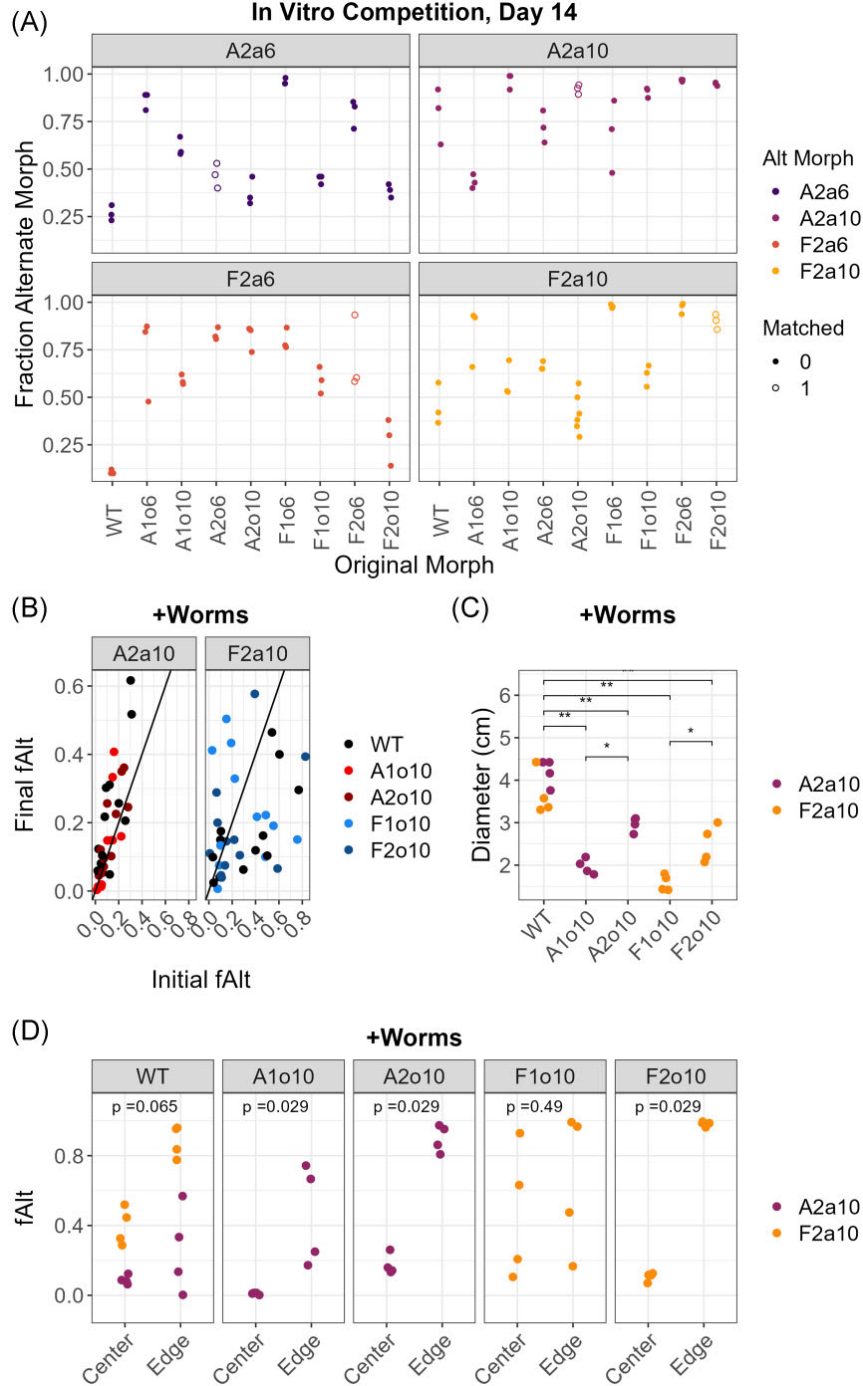
Alternate morphs of *C. gleum* coexist in pairwise competitions with original morphs. (A) Fraction of alternate morphs *in vitro* in coculture with original morphs on 6 cm 1.5% NGM agar plates. Plates were handled identically to those in experimental evolution (see the section “Materials and methods”). Ancestral *C. gleum* (WT) is used as a reference. Data are fraction alternate morph (fAlt) after two passages (14 days) in pairwise coculture (time series in Figure S5, Supporting Information). Empty symbols indicate pairs where alternate and original morphologies are matched (from the same community, replicate, and passage, e.g. A2o6 and A2a6). (B)–(D) fAlt morphs in the presence of N2 *C. elegans*. Experiments were conducted as in (A) except that populations were initiated with a range of initial fAlt (20%–50%) and ∼10 gravid hermaphrodites were added to each plate on day 0. (B) Summary of population trajectories in fAlt morph. Each point represents one pair of measurements at the start and end of a 7-day passage (days 0–7 and days 7–14). The 1:1 line (solid line) indicates ecologically stable population structure; when a population is on this line, the frequency of the alternate morph is the same at the start (initial) and end (final) of a passage. (C) Diameter (cm) of *C. gleum* colonies on worm+ plates (1.5% NGM agar) 3 days after inoculation. Tests are Wilcoxon rank-sum for indicated pairs (*, *P* < .05; **, *P* < .01). (D) Fraction of the alternate morph at the center (near the inoculation point) and at the edge of 7-day worm+ NGM plates. Tests are Wilcoxon rank-sum for fAlt morph at plate center vs. edge.

Coexistence was maintained in all pairs of alternate and original morphs (Fig. 2A). Populations were similar at the end of the first (day 7) and second (day 14) passages for most combinations (Figure S5, Supporting Information) and extending a subset of conditions for one additional passage had no further effect on ratios or abundances (not shown), suggesting that a local ecological equilibrium had been reached for most pairs.

The final fraction of alternate morph (fAlt) varied across pairs (10%–99% at day 14), but some trends were evident (Fig. 2A; Table S1, Supporting Information). The ancestral original-morph strain (WT) generally showed higher relative abundance (lower fAlt) than evolved original morphs (beta regression with fAlt as dependent variable, *P* ≈ 5.8e^−6^). Alternate morphs from the final passage tended to show higher relative abundance than passage-6 isolates (*P* ≈ 1.3e^−5^). Community of origin (A1/A2/F1/F2) did not predict outcomes, and alternate morphs did not consistently perform differently in competition with original morphs from the same community and passage, as would be expected with Red Queen-type arms-race dynamics (all indicated terms not significant after correction) (Morran et al. 2011). Qualitatively, across strains and pairs, we observed that alternate morphs tended to increase from rare (fAlt < 10%) but rarely decreased from common (> 80%). This indicated that intraspecies competitive exclusion does not explain the absence of alternate morphs in communities A1 and F1.

Much of the variation in outcomes remained unexplained. A model using strain IDs as independent variables for fAlt (beta regression pseudo-*R*^2^ = 0.43 with 13 terms) performed somewhat better than the model using community + passage (pseudo-*R*^2^ = 0.32 with eight terms), while an extended model using strains and combinations (37 terms) accounted for much of the variation in fAlt (pseudo-*R*^2^ = 0.91) (Table S1, Supporting Information), suggesting that pair-specific interactions were important for describing this variation. We, therefore sought to determine whether differences in growth parameters within competing pairs could explain this variability. To do so, we compared differences in growth rate (r_Alt_–r_Ori_) and differences in saturation log_10_(CFU/ml) in NGM medium (K_Alt_–K_Ori_) against fAlt in *in vitro* pairwise competitions for all pairs of isolates. We observed no correlation between growth parameters and outcomes in pairs (Figure S6, Supporting Information).

#### Pairwise competition on worm plates

These *in vitro* results differed from those observed during experimental evolution. Alternate morphs were present at higher relative abundance during *in vitro* pairwise competition (Fig. 2) than in the original samples (Fig. 1). In many pairs, the alternate morph was > 50% of the population (maximum alternate morph ∼99%), as compared with a maximum of ∼30% alternate morph during experimental evolution.

As the presence of the worm had a marked effect on community structure (Fig. 1), we hypothesized that the worm might also alter interactions between morphs. We, therefore repeated pairwise competitions with *C. elegans* on plates to determine whether the worm could alter the relative abundance of alternate morphs. Pairs of alternate and original morphs were mixed to create a range of initial frequencies of the alternate morph, then spotted onto standard 6 cm NGM+OP50 plates as before, but with the addition of ∼10 gravid N2 hermaphrodites to each plate. Here, we used only CG from the final passage, representing presumably the most adapted isolates from these experiments.

The presence of worms altered *C. gleum* populations. In the presence of worms, fraction alternate morph (fAlt) was generally lower than in the absence of worms, with a stable point around 10% alternate morph across all pairs (Fig. 2B). For community A2 alternate morphs, points at higher initial fAlt fell above the 1:1 line, suggesting a second equilibrium at a fAlt above the range of these data; this is consistent with *in vitro* results where these isolates frequently comprised 90%+ of the population (Fig. 2A). For community F2 alternate morphs, the data suggested a second unstable equilibrium around fAlt = 0.4.

Further, the presence of worms altered the spread of *C. gleum* over the agar surface. Unlike worm-free plates where CG grew only around the area of the initial spot, CG lawns eventually covered the plate surface when worms were present. Day-3 population diameter differed across original morphs (Kruskal–Wallace *P* = 2.8e^−4^, df = 4) and was greatest on plates with the ancestral isolate and lowest on plates where alternate morphs were competed against nonmatched evolved original morphs (A1o10 vs. A2a10, F1o10 vs. F2a10; Fig. 2C).

We hypothesized that alternate and original morphs might play different roles in worm-dependent population expansion on these plates. If so, the prevalence of alternate morphs at the edge of the plate (farthest point of population expansion) should be different than at the site of the original inoculum. To determine the role of alternate morphs in expansion of *C. gleum* populations on worm plates, we measured population composition at the plate center (near the original inoculum) and the plate edge after 7 days. In most pairs, alternate morphs were over-represented at the plate edge relative to the center, and the magnitude of the over-representation was greatest in matched pairs of original and alternate morphs (A2o10 vs. A2a10, F2o10 vs. F2a10) (Fig. 2D).

#### Motility

**Figure 3.**
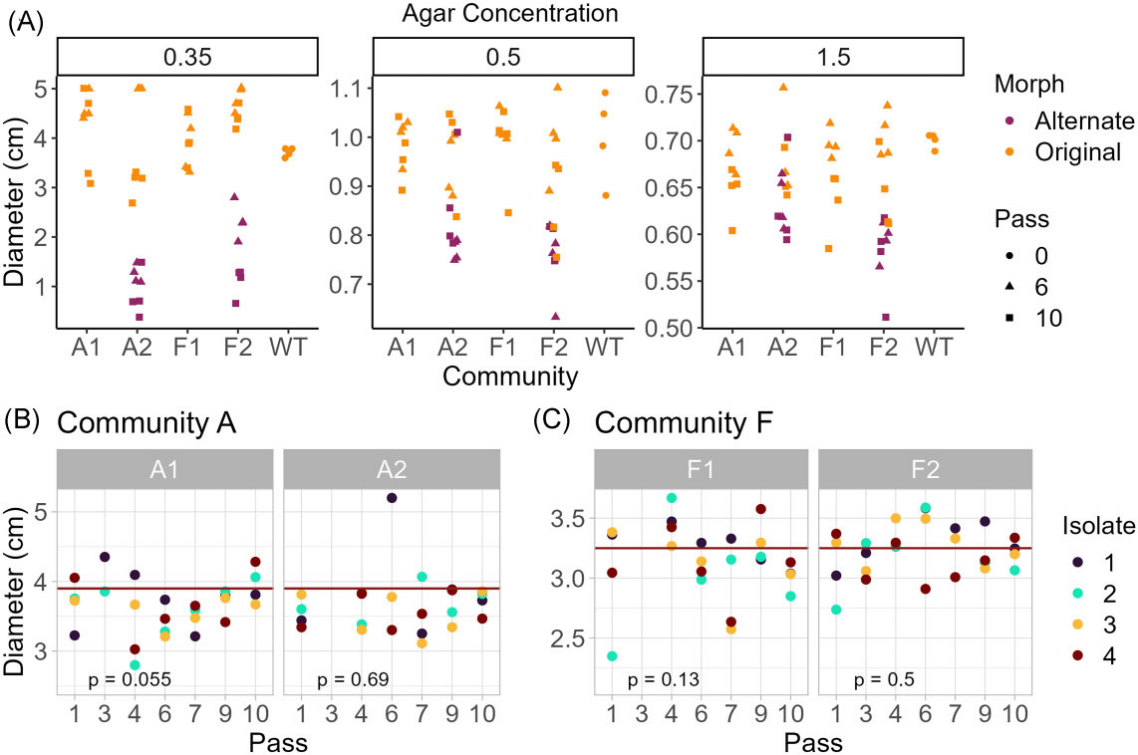
Surface motility of original and alternate morphs of *C. gleum*. (A) Motility on 0.35%, 0.5%, and 1.5% NGM agar. Colony diameter (cm) is the *y*-axis and community ID is on the *x*-axis. “Pass 0” is represented by the ancestral WT. Each shape represents one community+replicate (*n* = 4 isolates from single colony picks for each combination of community+replicate+passage). (B) and (C) Surface motility of original morphs from passages 1 to 10 of communities (B)-A and (C)-F, on soft 0.35% NGM agar (*n* = 4 isolates per community+passage). Isolates from passages 6 and 10 are the same as in panel (A); missing data reflect passages and communities for which colonies could not be retrieved from glycerol stocks. The horizontal line indicates the median diameter of ancestral WT isolates (*n* = 4) from the same day. Tests are Kruskal–Wallace of motility diameter at ∼1.8 days vs. passage. Note that community-A and community-F isolates were assayed on separate days with separate batches of freshly prepared plates, and the raw diameters cannot be compared across days due to inherent variability in these assays.

As motility of community members was altered in an earlier, similar experiment (Taylor et al. 2022), we next sought to determine whether morphotypes could be characterized by differences in this trait. *C. gleum* lacks flagellar motility (Steinberg and Burd 2015), but like many other *Flavobacteriales*, shows gliding motility on the surface of soft (0.35%) agar plates (McBride and Zhu 2013, Shrivastava et al. 2013, McBride 2014, Sato et al. 2021). Surface motility assays were therefore conducted on soft (0.35%), swarming (0.5%), and solid (1.5%) NGM agar to determine whether the alternate morphology was associated with changes in motility (Fig. 3). All isolates were motile on soft agar, and alternate morphs were consistently less motile than original morphs (Fig. 3A; Wilcoxon tests of original vs. alternate morphs: 0.35% agar, *P* = 1.47e^−8^; 0.5% agar, *P* = 5.65e^−7^; and 1.5% agar, *P* = 5.84e^−6^). Early (pass 6) and late (pass 10) isolates of each morphotype did not differ in motility (all comparisons not significant after correction) and we did not observe any trend in motility over passages (Fig. 3B). These data do not indicate directional selection on motility overall. Further, these results cannot explain enrichment of alternate morphs at the population frontier on standard (1.5% agar) plates with *C. elegans*, as alternate morph colonies spread less even on this firm substrate.

#### Sequencing

We next sought to identify genetic differences that would provide insight into the phenotypic differences between alternate and original morphs. Eleven strains (Ancestor, A1o10, A2o6, A2a6, A2o10, A2a10, F1o10, F2o6, F2a6, F2o10, and F2a10) were sequenced to identify genomic changes during selection. Overall, we observed very little genetic divergence between isolates (Table S2, Supporting Information). All of the putative “SNPs” identified in our initial analysis mapped to low-complexity regions and/or the ends of contigs (Table S2, Supporting Information) and may, therefore, represent sequencing or alignment errors rather than real sequence differences. No putative mutations were unique to and conserved over alternate morphs.

*Chryseobacterium* and other Flavobacteriales show gliding motility (McBride and Zhu 2013, Gavriilidou et al. 2020), which depends on a type 9 secretion system (T9SS) (Trivedi et al. 2022). All sequences showed complete sets of homologs to known components of gliding motility, specifically SprA, PorV, PorP, PorT, and all *gld* genes. As is typical for this clade, *gldN, gldM, gldL*, and *gldK* are adjacent to one another and appear to be a single operon; the same is true for *porV, porU*, and *gldJ*, and for *gldB* and *gldC*. We did not find homologs of SprB or other secreted adhesins in these genomes; BLAST search of the *F. johnsoniae* operon *sprCDB* (GenBank EF111026.1) against *Chryseobacterium* generated no hits, indicating that the genus in general does not have the SprB adhesin. These *C. gleum* genomes do contain an annotated LolA outer membrane lipoprotein, which can be used for spreading on soft agar in the absence of adhesin activity (Sato et al. 2021); as with other motility genes, no SNPs were observed in the *lolA* sequence or adjacent regions.

When compared with the ancestral genome, no SNPs were observed in known motility or secretion genes or in regulatory regions associated with any of these genes in any evolved isolates. The lack of genetic differences between original and alternate morphologies in motility encoding regions indicated that differences between morphotypes did not arise from simple loss or modification of these components. This aligns with previous findings in other Flavobacteriales, where altered morphologies associated with reduced motility were not linked to mutations in motility genes (Penttinen et al. 2018).

#### Phenotype *×* environment for alternate and original morphs

We next sought to characterize differences between isolate phenotypes by changing the environment. Concentration of a rich nutrient source (e.g. peptone) has been shown to alter motility in *Chryseobacterium* and related Flavobacteriales (Gavriilidou et al. 2020, Sato et al. 2021, Khare et al. 2022). This suggested that differences in the relationship between motility and nutrient levels in original vs. alternate morphs could be used to gain further insight into the differences between morphotypes.

We, therefore assessed motility on 0.35% NGM agar where nutrient concentration was altered by changing the concentration of peptone (0.2X, 1X, or 4X of the standard formulation). NGM contains peptone at a standard concentration of 2.5 g/l, compared with Difco LB medium at 10 g/l tryptone; 4X NGM is, therefore equivalent to 1X Difco LB in peptone-equivalent concentration. The range of NGM peptone concentrations used here recapitulates the range of LB concentrations over which earlier studies showed changes in motility in *Chryseobacteria*.

**Figure 4.**
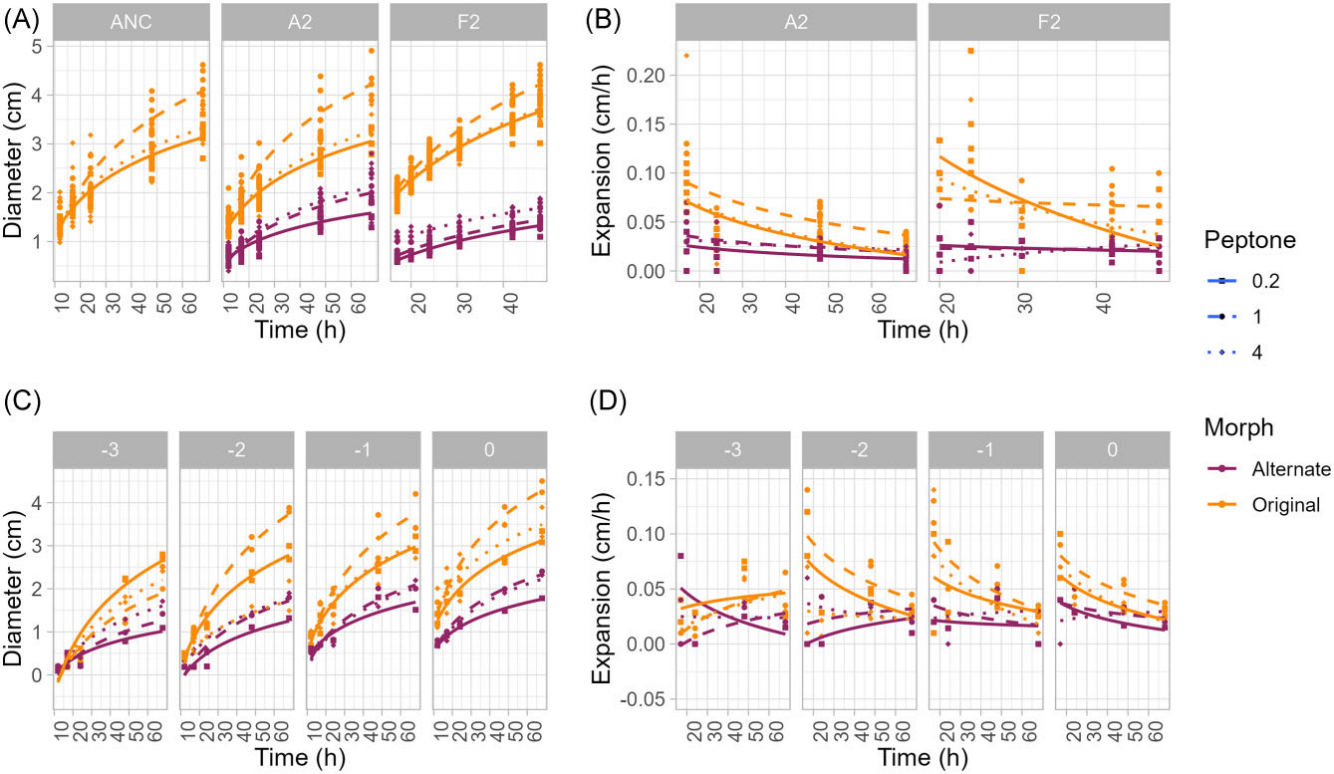
Nutrient concentration alters *C. gleum* surface motility on NGM agar. (A) and (B) Here, nutrient concentration was altered by changing the concentration of peptone in 0.35% NGM agar (0.2X, squares + solid lines; 1X, circles + dashed lines; or 4X, triangles + dotted lines) while holding all other components constant. Strains in these experiments were alternate and original morph isolates from the ancestral strain and from communities A2 and F2 pass 6 (*n* = 3 isolates per morph, technical triplicate; all data points shown). Isolates from communities A and F were assayed in separate runs; ancestor was run with community A. (A) Diameter (in cm) over time. (B) Expansion rates (cm/h) calculated from the data in (A). (C) and (D) Colony diameters (C) and expansion rates (D) for community A2 pass 6 isolates (*n* = 3 isolates per morph) across a range of dilutions of the original inoculum, on NGM agar plates with varying peptone concentrations (0.2–4X, top axis). Dilution 0 represents the inoculum used in (A) and (B) (1 µl inoculum containing ∼10^6^ CFU), from which the indicated series of 10-fold dilutions were made (lowest dilution contained ∼10^3^ CFU/µl). Lines show fits to data (y∼ ln(x)).

Motility in these assays had a weak but significant dependence on peptone concentration, and a strong and significant dependence on morphology of the isolate (Fig. 4A; Table S3, Supporting Information). In all regressions, community (A2 vs. F2) was a small but significant factor; however, as isolates from different communities were assayed on different days, this may be a batch effect. Consistent with expectations, original morphs were more motile than alternate morphs across all conditions (Table S3, Supporting Information). An intermediate level of peptone (1X) produced the greatest motility in original morphs (linear regression, diameter ∼ log(hours) + Peptone + Community, β_Peptone1X, ORI_=+0.41 *P* < 2e−^16^; β_Peptone4X, ORI_ not significant). Whereas original morphs showed expansion rates which declined over time (linear regression, [expansion rate] ∼ log(hours) + Peptone + Community, β_ln(time), ORI_= −0.04, *P*<2e−^16^), alternate morphs expanded at effectively fixed rates (β_ln(time), ALT_ and β_Peptone, ALT_ not significantly different from zero, all *P* > .05) (Fig. 4B; Table S3, Supporting Information). Additionally, morphology of the colony frontier, and particularly the branching pattern, was altered by peptone concentration (Figure S7, Supporting Information) and was specific to morphotype.

Flavobacteriaceae are known to show density-dependent and density-independent phases of gliding motility (Álvarez et al. 2006, Penttinen et al. 2018, Gavriilidou et al. 2020, Sato et al. 2021, Khare et al. 2022). We hypothesized that the time-dependence of expansion rate in original morphs, and the lack thereof in alternate morphs, was related to density-dependence of motility. Specifically, we hypothesized that expansion rates in original morphs should show dependence on initial inoculum density, whereas this interaction should not occur for alternate morphs. To test this, motility plates were established with inoculum densities across four orders of magnitude (Fig. 4C and D). The relationship between surface motility (diameter) and inoculum density differed between alternate and original morphs (Fig. 4C, likelihood ratio test (LRT) *P* < 2.2e^−16^ for nested linear regressions with vs without morph as a predictor). For alternate morphs, inoculum density altered diameter early in expansion [intercept in linear mixed effects model (LME), CI for all terms excluded 0] but not expansion rate (LRT *P* = .92). Inoculum density had a larger effect on early time diameter for original than alternate morphs (LME coefficients for alternate vs original morphs: dilution −3, intercept −2.2 vs. −3.6; dilution −2, −2.1 vs. −3.1; and dilution −1, −1.7 vs. −2.6). Overall, expansion rate of original morphs was not significantly affected by inoculum density (LRT *P* = .09), but the lowest dilution (−3) is significantly different from the rest of the data (*P* = .012), indicating a threshold for density dependence. This is true particularly at early times, when higher-density inocula show a phase of rapid expansion (Fig. 4D).

Peptone concentration significantly affected both expansion rates on soft agar (LRT *P* = 1.1e^−15^) and overall biomass productivity (Figure S8A and D, Supporting Information) in both morphs. However, the observed motility differences between morphotypes were not explained by differences in growth parameters (Figure S8, Supporting Information). Even in nutrient conditions where original and alternate morphs showed indistinguishable growth rates and/or carrying capacities, the original morph was substantially more motile. This indicated that morphotype differences in the phenotype x environment interaction for motility could not be explained simply by differences in cell density or rate of production of new cells.

## Discussion

In this study, we observed community-based evolution in microbial consortia on plates with *C. elegans*. The presence of the worm altered community composition and the evolution of member taxa, with intraspecies morphological diversity emerging in worm-associated communities. Here, we focused on characterization of diversity in *C. gleum*, which developed an alternate morphotype in one replicate of each of two communities (A and F). Alternate morphs from different communities were phenotypically almost indistinguishable, indicating convergent evolution of intraspecies diversity in *C. gleum*. Emergence of the alternate morphotype was rare, but intraspecies diversity was robustly maintained. Spatial population expansion on plates, apparently with *C. elegans* as a vector, favored maintenance of the alternate morph.

The selective processes that resulted in diversification are not fully clear. It has been suggested that interspecific niche-based competition can promote diversification of focal species in microbial communities (Chu et al. 2021). Consistent with this idea, we did not observe alternate morphotypes during evolution of bacterial monocultures with worms during a similar experiment (Taylor et al. 2022). Alternate morphs of *C. gleum* emerged at a low point in community diversity (Figure S2, Supporting Information), consistent with observations that low-diversity communities are more likely to generate new diversity due to niche availability (Madi et al. 2020, 2022).

In a structured environment, colonization of empty space is a component of niche competition; spatial expansion may easily be important for both inter- and intraspecies niche-based interactions. Understanding interactions among member species is, therefore of interest for understanding community-level outcomes. However, *C. gleum* morphs coexisted in single-species populations, indicating that the multispecies community was not necessary for maintenance of diversity. Spatial structure on plates may be sufficient to explain coexistence (Lowery and Ursell 2019, Gude et al. 2020, Wu et al. 2022). It is also plausible that our alternate morph isolates are not first-step mutants, and that propagation of early rare variants did require the community context. Additionally, there may be frequency- or density-dependent interactions outside the range of our assays that might explain the importance of the community.

From first principles, it is likely that selection in *C. gleum* occurred mostly in the environment rather than in the worm host. *C. gleum* is a pathogen, causing substantial mortality in adult N2 worms within 1 day of exposure (Figure S3, Supporting Information); in fact, colonization by these bacteria is difficult to measure due to mortality. Community plates with *C. gleum* supported small, struggling populations of worms and heavy lawns, suggesting pathogen-related mortality. Despite high relative abundance of *C. gleum*, total worm-associated populations of this bacteria were small, generally < 10^3^ bacteria per adult worm. Further, nearly all worms on plates toward the end of a passage were juveniles, which are expected to support smaller intestinal bacterial loads than adults (Portal-Celhay et al. 2012). This indicates that *C. gleum* populations in the collective intestines of worms on these plates were small and short-term and suggests that *C. gleum* was maintained largely in the environment rather than in the host.

In this context, the worm can act as both a predator and a vector. The worm is well known to spread bacteria by carriage in the gut and on the external cuticle (Kenney et al. 2005, Thutupalli et al. 2017, Bermudez et al. 2018). Alternate morphs had an advantage in expansion on hard agar plates only when *C. elegans* were present, suggesting that vectoring of bacteria by the worm is relevant. It is plausible that alternate morphs of *C. gleum* were selected for during spatial expansion; as *C. gleum* gliding motility is ineffective on hard agar, this expansion required use of a nematode vehicle. This is the only case where coevolution of alternate and original morphs (same community and replicate) affected the outcomes of intraspecies competition.

However, it is not clear what trait(s) of the alternate morph are relevant. Gliding motility is essentially ineffective on hard (1.5%) agar, and the alternate morph still shows smaller colonies than original morphs on hard agar. It is unlikely that the motility phenotype is directly relevant for spatial expansion here. Rather, these results may indicate selection on a trait that is coregulated with motility.

One possibility is biofilm formation. Mutants in lineages of motile biofilm-forming bacteria often show a tradeoff where increased motility is associated with decreased biofilm formation and vice versa (van Ditmarsch et al. 2013, Palma et al. 2022). The T9SS is important for both motility and biofilm formation in other Bacteroidota, and these phenotypes are linked (Penttinen et al. 2018, Eckroat et al. 2021). Changes in aggregation or biofilm formation might affect the ability of bacterial cells to adhere to the worm cuticle, to be ingested by the worm, and/or to survive passage through the intestine. Measuring biofilm formation and vectoring by the worm, and determining the bacterial traits that affect vectoring, remain a challenge for future work.

Although the motility phenotypes do not explain expansion of *C. gleum* on hard agar plates, and as such are used here only as an experimentally accessible indicator of differences between morphotypes, our observations are interesting as a source of insights into T9SS-dependent motility. Our specific results were inconsistent with other work where *Chryseobacterium* motility increased as the concentration of rich medium (LB or Shieh) was decreased (Penttinen et al. 2018, Khare et al. 2022). Further, our results are in contrast with other work (Khare et al. 2022) where high tryptone concentrations produced a smooth motility frontier in *Chryseobacterium*. The authors suggested that this was a result of higher cell densities at high nutrient levels. We observed a nearly smooth frontier at low peptone for all original morphs, whereas high peptone produces dense growth and a heavily branched frontier (Figure S8, Supporting Information), suggesting that the structure of the expansion frontier is due to collective behavior rather than density alone.

These observations, together with our observation that an intermediate concentration of peptone maximized expansion of original morphs, suggest that the properties of the spreading frontier are controlled by a combination of individual and collective motility (Patra et al. 2016) and that use of these modes differs between morphs and across environments. From theory, we expect that branching will be promoted when directional movement of individual cells (e.g. taxis into unoccupied space) is relatively low (Giverso et al. 2015, Bisht et al. 2017). Further, expansion rate should be maximized at some value of individual motility (Bisht et al. 2017), which could explain why original morphs showed higher motility at 1X peptone than at 0.2X or 4X. It is plausible that differences in directional movement and growth across strains and environments are sufficient to explain these results; this remains to be determined. Aggregate formation may be important as well, particularly for understanding changes in branching structure (Li et al. 2021).

While evolution often produces compositionally similar communities, individual microbial lineages exhibit distinct evolutionary trajectories within these communities. Adaptation in real (or realistic) environments, with spatial structure and interspecies/interkingdom interactions, tends to select for variants that interact differently with physical space and with other biological agents (Hansen et al. 2007, Santos-Lopez et al. 2019). Although growth rates and carrying capacities are historically used as part of definitions of “fitness” in the laboratory, real world fitness of microbes is often weakly associated with these parameters (Concepción-Acevedo et al. 2015, Momeni et al. 2017, Bansept et al. 2021). Instead, fitness in a spatially structured environment with biotic interactions is more often a function of physical and social interactions, where spatial scales and interaction structures are critical aspects of selection (Hansen et al. 2007, France et al. 2018, Liu et al. 2019, Gorter et al. 2020, Conwill et al. 2022, Hoces et al. 2022). Investigations of realistically structured populations, where temporal and spatial dynamics are considered explicitly, are important for understanding microbial evolution and intraspecies diversity.

## AI usage statement

During the preparation of this work, the authors used ResearchRabbit.ai to accelerate literature search and ChatGPT to revise portions of the text for clarity. After using these tools/services, the authors reviewed and edited the content as needed and take full responsibility for the content of the publication.

## Supplementary Material

fiae039_Supplemental_Files

## Data Availability

All data and code used in this manuscript are available at https://github.com/veganm/NRRL_CG_Motility. Sequence data are available from NCBI BioProject PRJNA764097.
