# Supplementary material for: Parallel evolution of alternate morphotypes of Chryseobacterium gleum during experimental evolution with Caenorhabditis elegans
Source: FEMS Microbiol Ecol. 2024 Mar 28;100(5):fiae039. doi: 10.1093/femsec/fiae039 (PMC11004935; doi:10.1093/femsec/fiae039)
Supplement: fiae039_Supplemental_Files [file fiae039_supplemental_files.zip › FEMS_Suppdata TableLegendRevised.docx]

Table S1. Models and parameter estimates for beta regressions on data from *in vitro* pairwise competitions among alternate and original morphs. Data are shown in Figure 2 and Figure S6.

Table S2. Full list of “SNPs” across sequenced genomes of *C. gleum* from communities A and F, relative to the ancestral genome. SNP identification was performed after multiple sequence alignment in Mauve.

Table S3. Coefficients of linear regression models for gliding motility on NGM with varying peptone levels (data in Figure 3). LRTs of the full model vs. reduced models with each individual factor removed supported use of the full model (all p<1e-15). Significance codes: 0 ‘***’ 0.001 ‘**’ 0.01 ‘*’ 0.05 ‘.’ 0.1 ‘ ’ 1
