# Supplementary material for: Parallel evolution of alternate morphotypes of Chryseobacterium gleum during experimental evolution with Caenorhabditis elegans
Source: FEMS Microbiol Ecol. 2024 Mar 28;100(5):fiae039. doi: 10.1093/femsec/fiae039 (PMC11004935; doi:10.1093/femsec/fiae039)
Supplement: fiae039_Supplemental_Files [file fiae039_supplemental_files.zip › Supp data SI_Vega_FEMS23_Revised.docx]

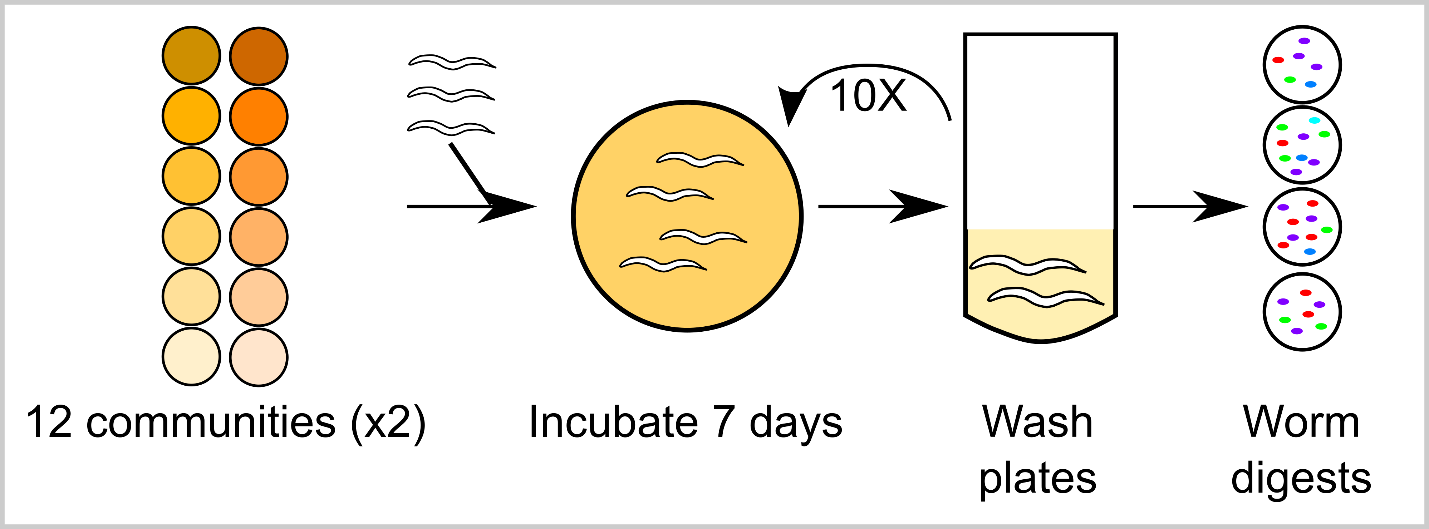


Figure S1. Experimental workflow for community-based evolution. The experimental set up consisted of 12 communities with seven members each, similar at a genus level but variable on a species/isolate level. Each community was initiated on two replicate plates (community A-L, replicates 1 and 2), to which L1 stage N2 *C. elegans* were added. These communities + worm populations were passaged every seven days to fresh NGM agar plates. At the end of each passage, adult worms were batched, physically disrupted to release gut contents, and plated in serial dilution to assess bacterial community composition. Unused volumes of worm batch digests were frozen in glycerol at –80°C to preserve a record of these communities, and single-colony isolates picked from worm digest plates were likewise frozen for further assays. Isolates reflective of original and alternate morphologies were used in phenotypic assays for growth, competition, and motility, and selected isolates were sent for whole-genome sequencing.


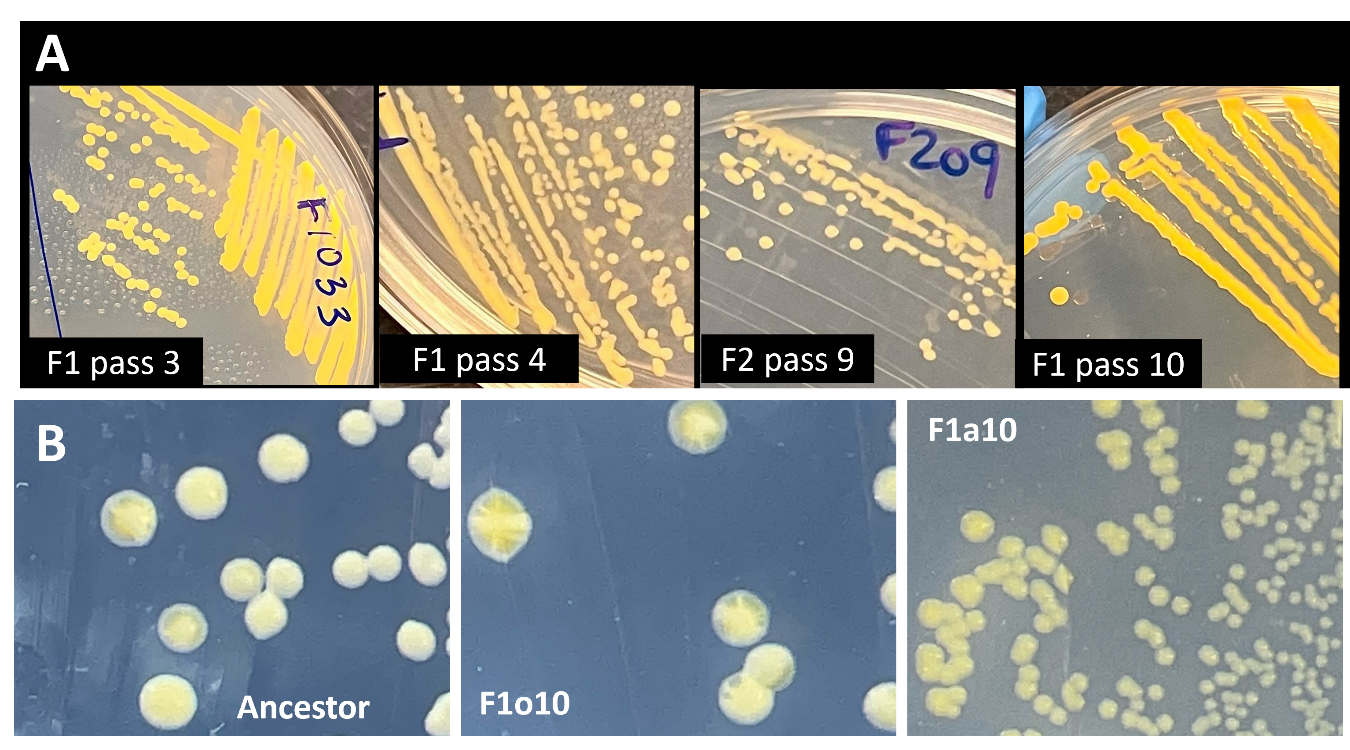


Figure S2. Variation in colony morphologies in *C. gleum.* (A) Variation in colony morphology on NA within the original morphotype in community F, multiple passages. Images are of NA plates after 48 hours at 25°C and were chosen to reflect the overall range of colony variation observed in these experiments. (B) Changing environmental conditions (here, NGM agar for 24 hours at 37°C) alters morphology, but distinctions between morphotypes (colony size, mucoidy) are conserved. Intensity of and differences in pigmentation are reduced on NGM, which has lower nutrient concentrations than NA.


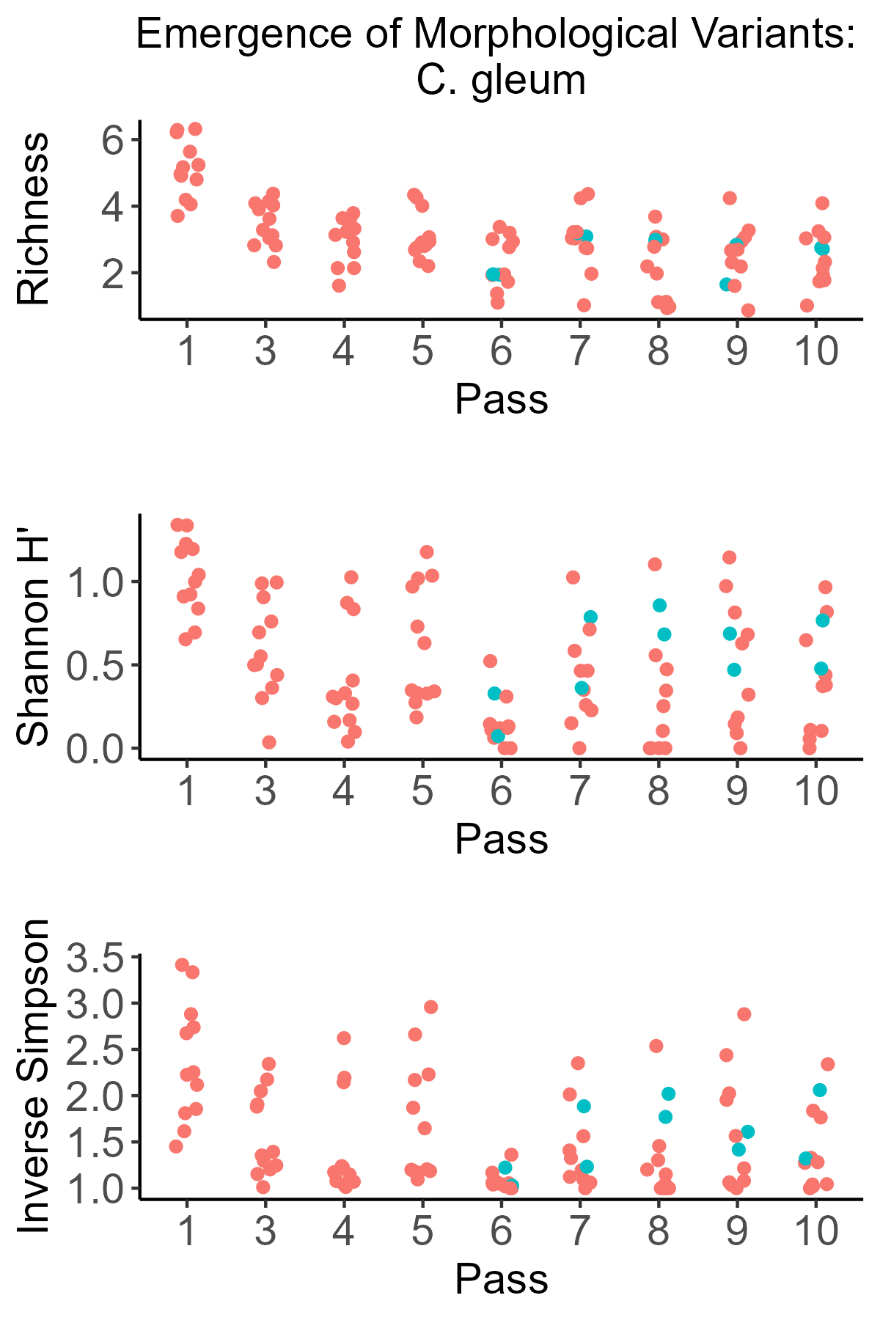


Figure S3. Emergence of alternate morphs of *C. gleum* coincides with time-series minimums of taxonomic diversity. Each data point represents one of two replicates of each of the 12 communities (n=24 points total at each passage). Communities without alternate morphs are shown in pink; communities with alternate morphs of the indicated species are shown in blue. Each row reports a different measure of community diversity (richness, Shannon H’, inverse Simpson).


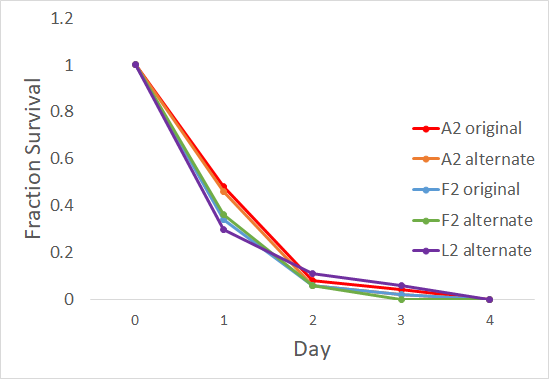


Figure S4. *Chryseobacterium* killing in N2 Bristol *C. elegans*. In these assays, day 2 synchronized adult worms (n=100) were added to 6 cm NGM plates with lawns of pass-10 isolates of *C. gleum* (A2, F2) or *C. indologenes* (L2) with original or alternate morphologies*.* Mortality was assessed at 24 hour intervals.


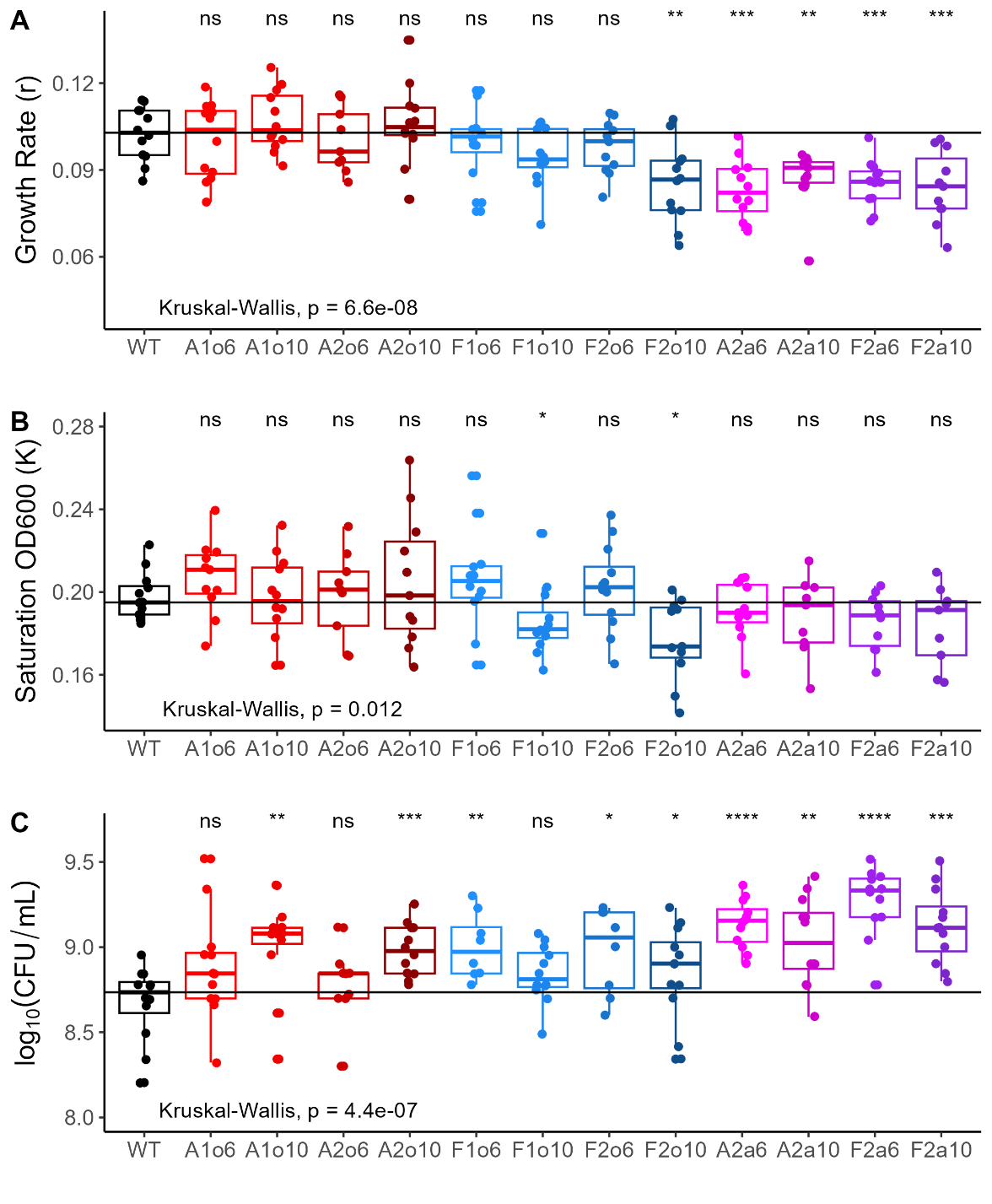


Figure S5. Phenotyping of *C. gleum* original (o) and alternate (a) morphs from communities A and F. Ancestral isolates are shown as “WT”. Maximum exponential growth rate (h^-1^) (A) and OD600 saturation density (B) were estimated from plate reader growth curves in liquid NGM media for 24 hours at 27°C. Saturation density in CFU/mL (C) was estimated by dilution plating the same cultures after growth in the plate reader. Data represent three independent experiments on separate days, using the same isolates in all runs (n=4 from each community and passage). Results of pairwise Wilcoxon tests vs. the ancestor are shown above each data set (*, p<=0.05; **, p<=0.01; ***, p<=0.001; ****, p<=0.0001).


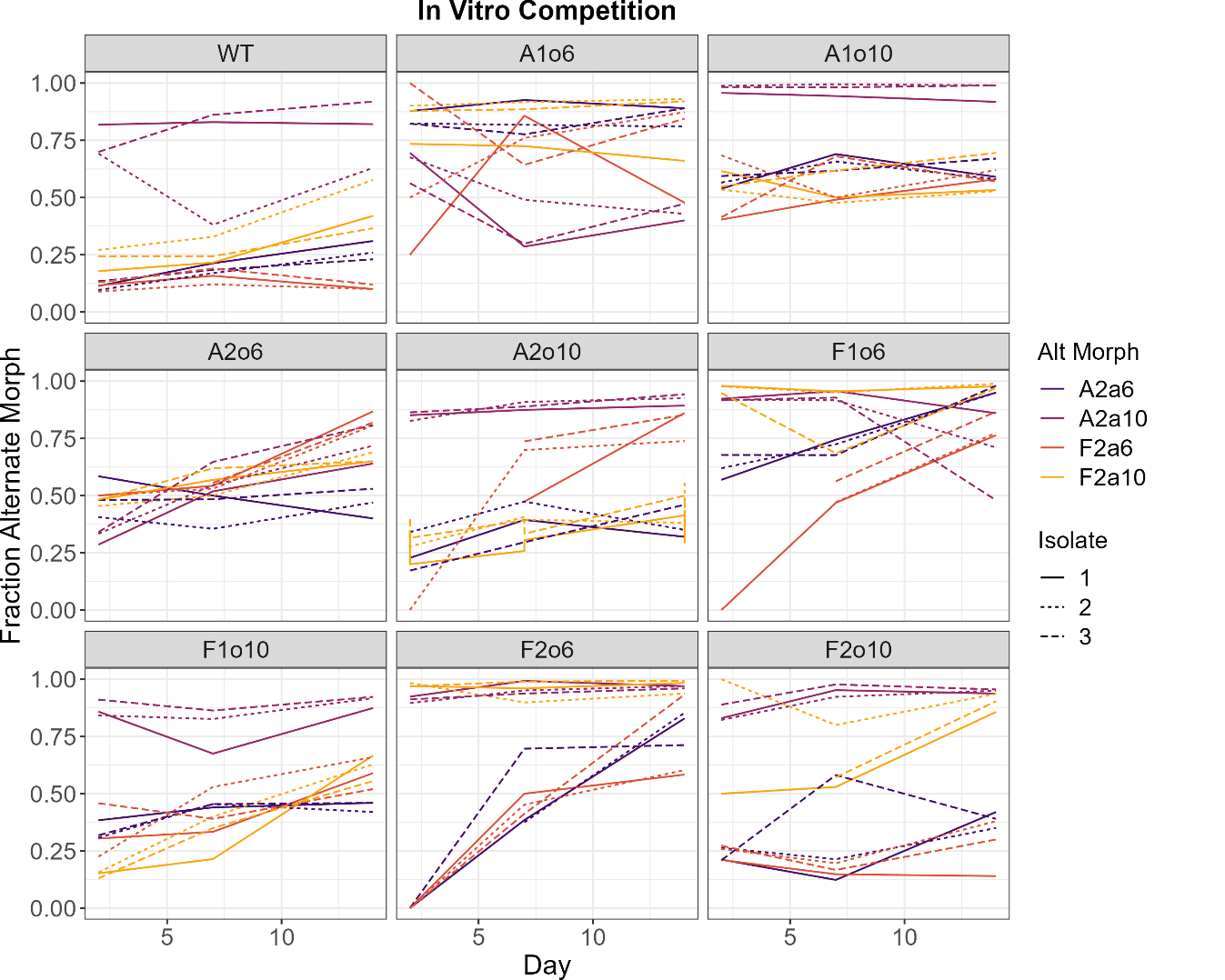


Figure S6. Trajectories of relative abundance of alternate morphology isolates, in pairwise competitions on standard NGM agar plates without *C. elegans*. Alternate morphs are represented by n=3 isolates from each community + passage; three isolates of each original morph were grown individually and pooled to create a common competitor for all pairwise competitions with that original morph.


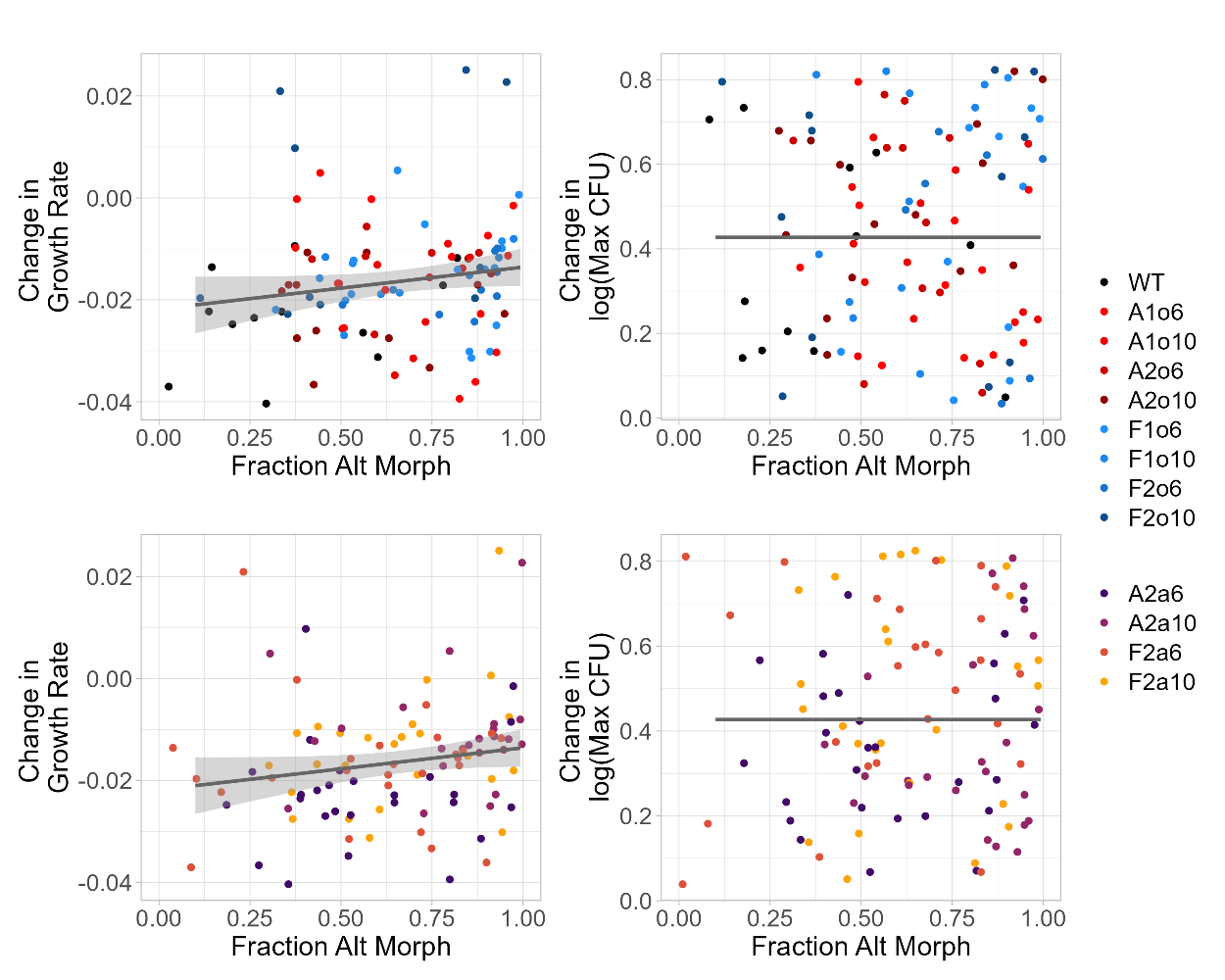


Figure S7. Neither growth rate nor carrying capacity in NGM explain outcomes of pairwise competition on plates without worms. Neither Δr (left) nor Δlog(CFU/mL) (right) between pairs of alternate and original morphs were significantly associated with fraction alternate morph in pairwise competition (linear regression slopes not significantly different from zero, p>0.05). Data are colored by original morph (top) and by alternate morph (bottom) to illustrate lack of dependence of these results on community and passage. Lines are linear regression fits to data; grey region is 95% CI.


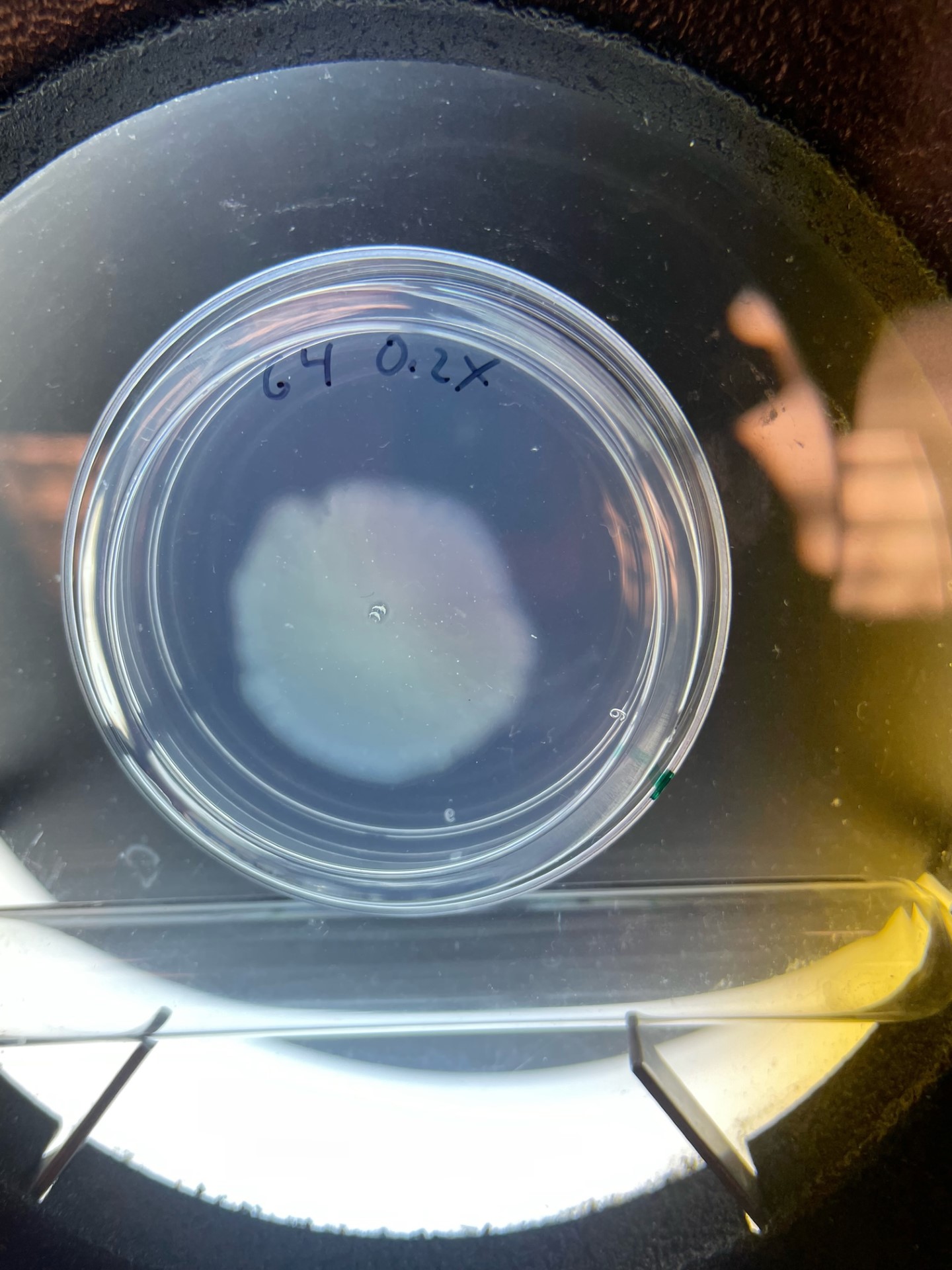

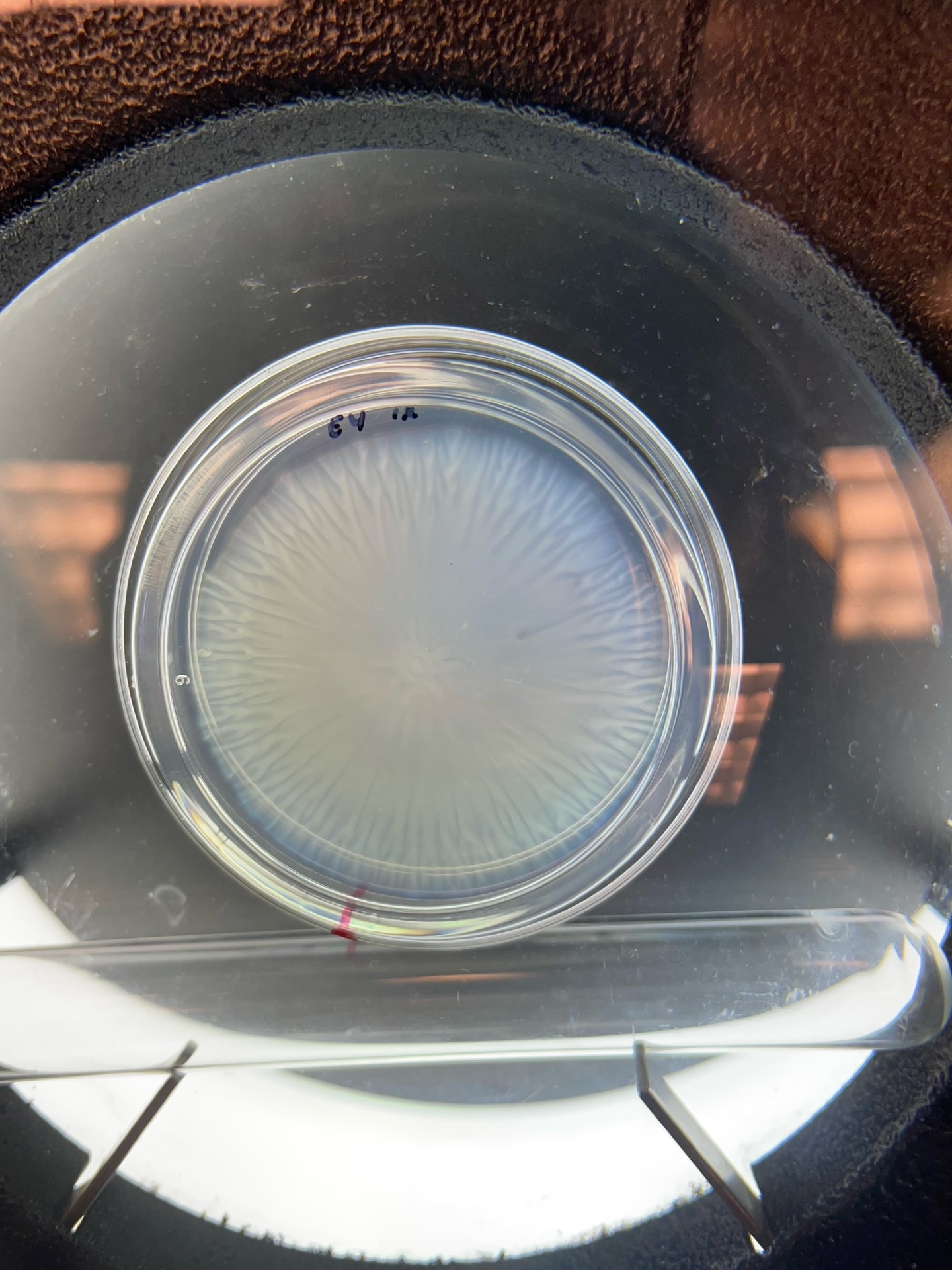

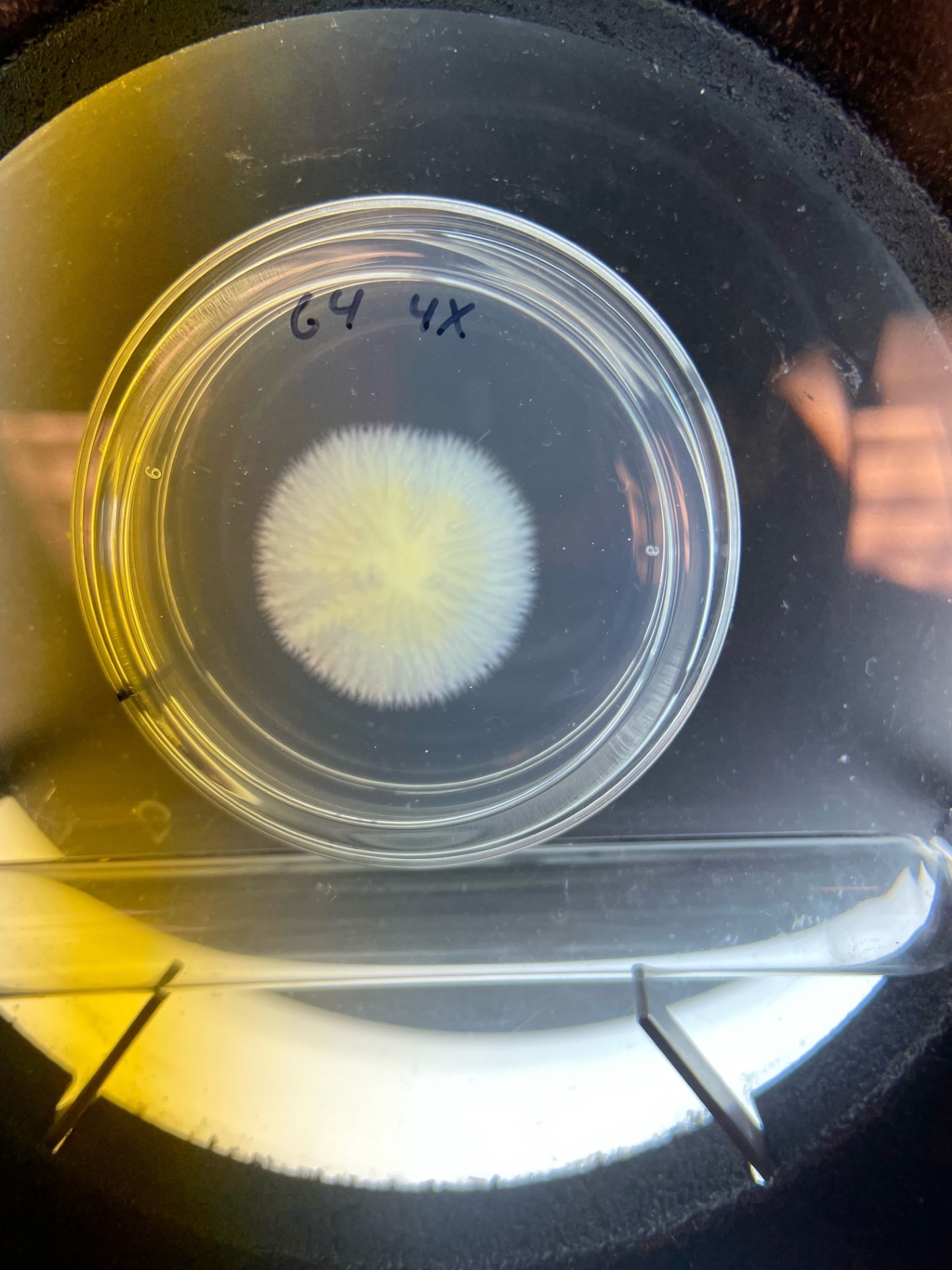

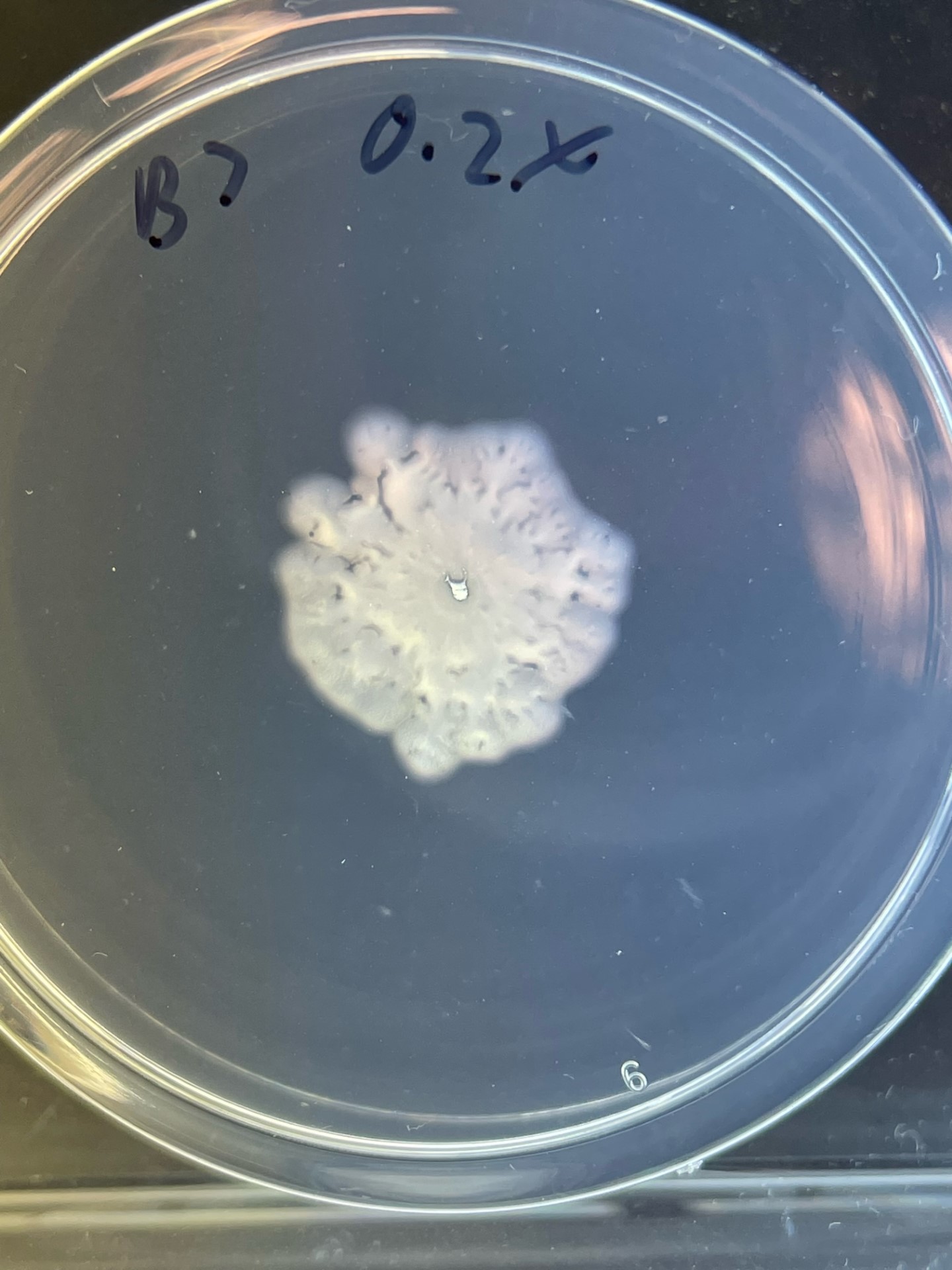

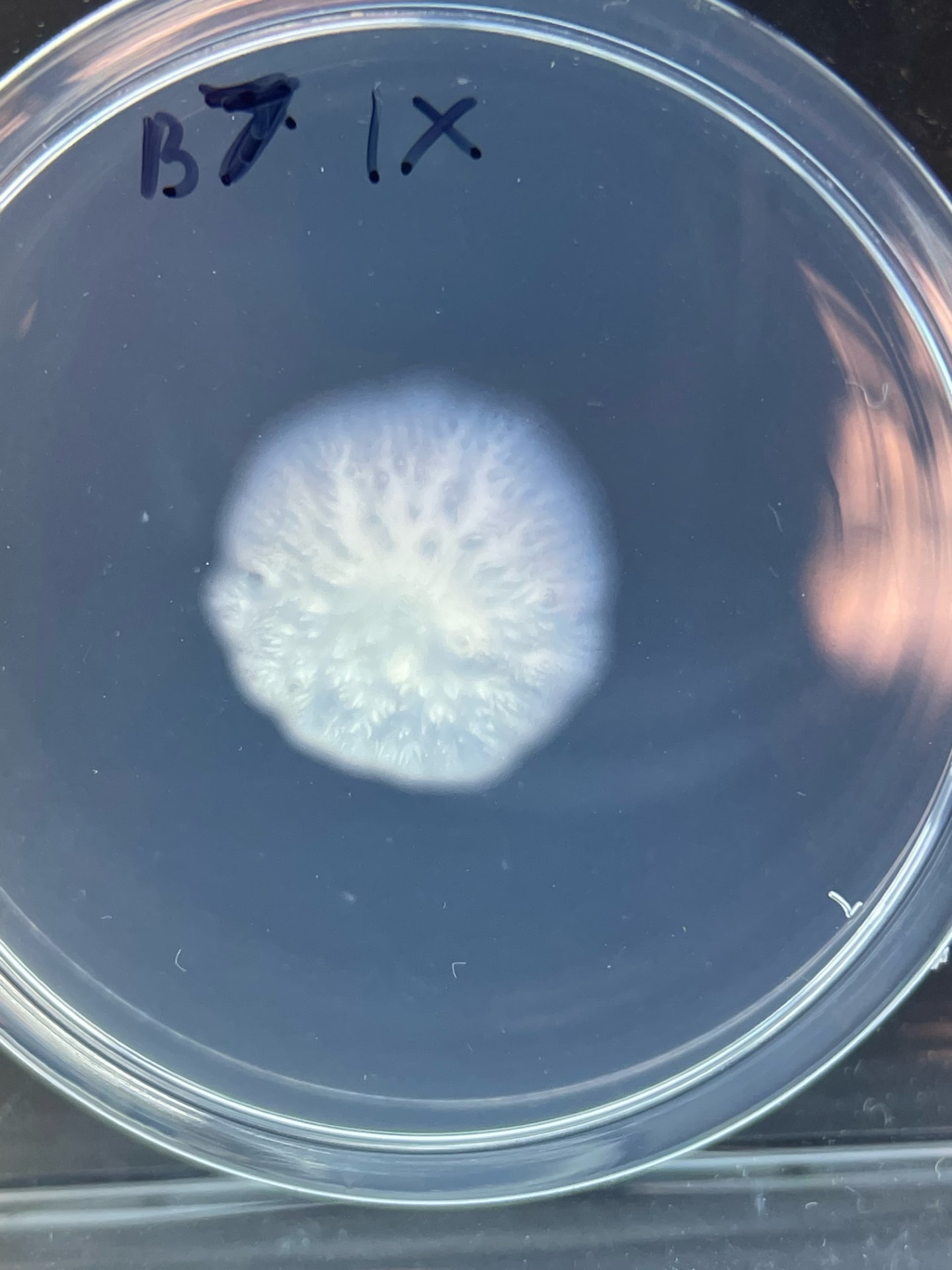

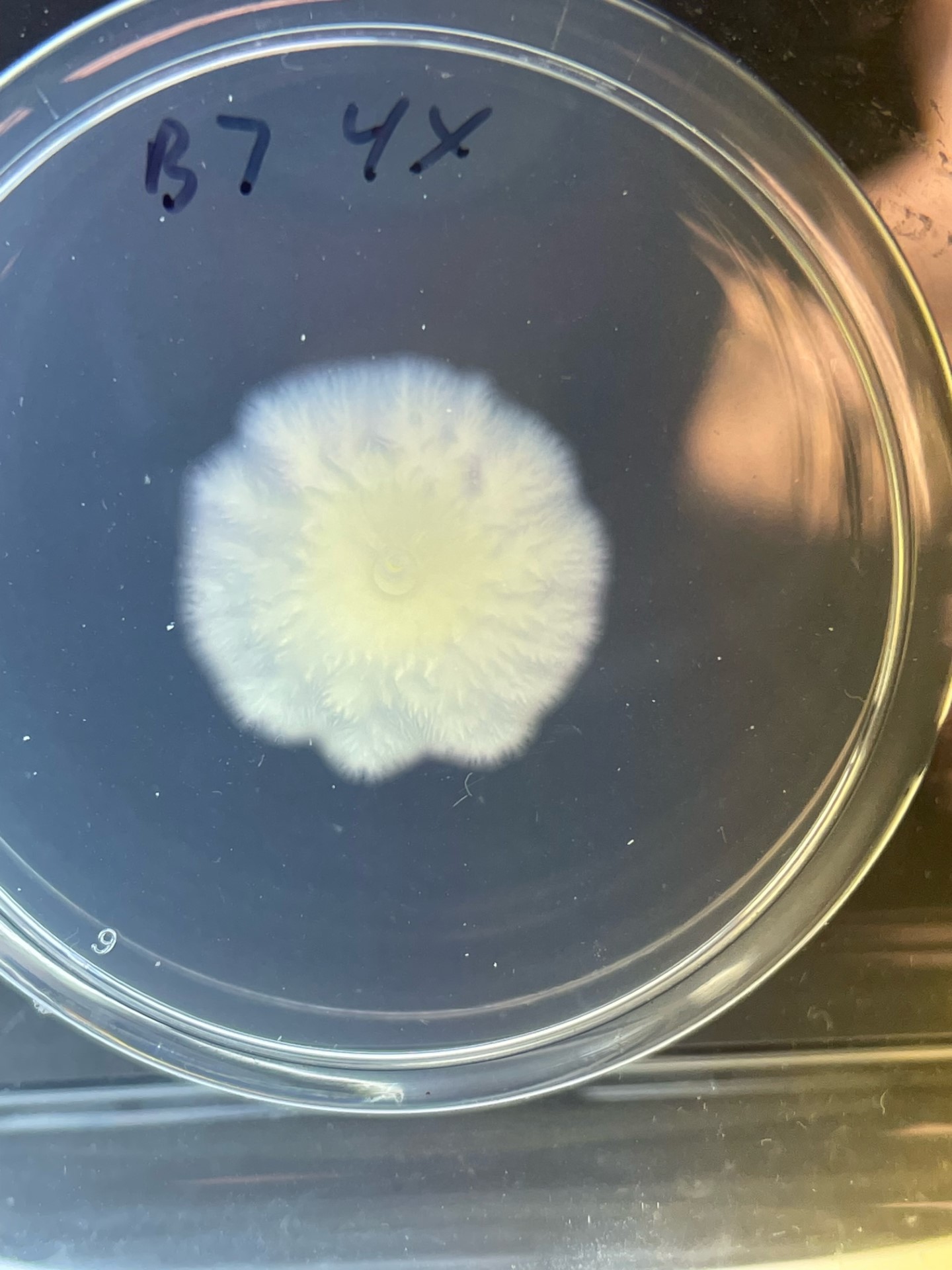


Alternate

Original

0.2X

1X

4X

Figure S8. Branching of the motility front on 0.35% NGM (72h) with difference concentrations of peptone, in an original morph (top, F2o10 isolate 3) and an alternate morph (bottom, A2a6 isolate 3). Images are zoomed in to show detail; diameters are not to scale. Plates shown are representative of each morphotype.


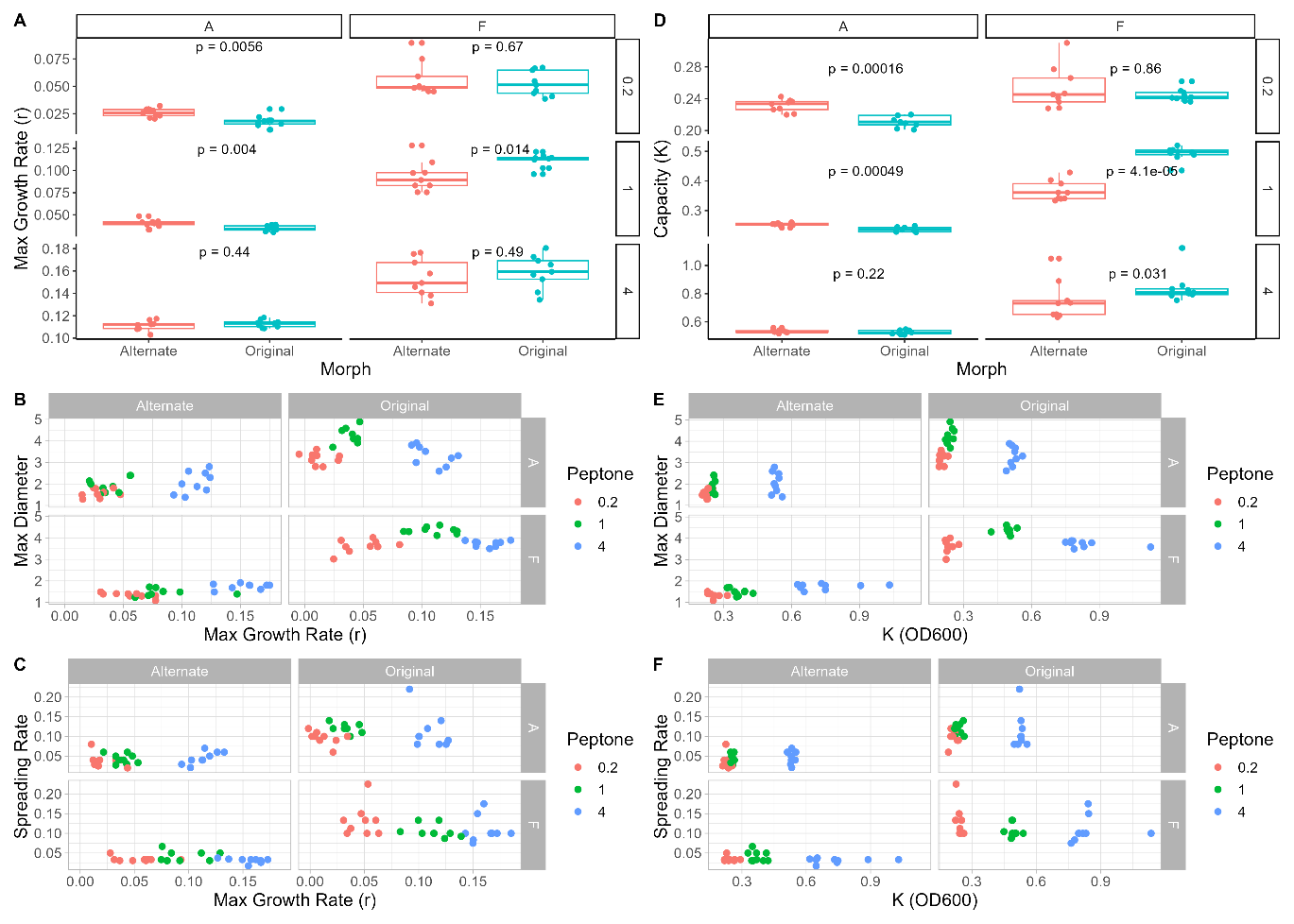


Figure S9. Nutrient concentration alter growth parameters, but growth parameters (growth rate *r*, A-C; carrying capacity *K*, D-F) do not explain differences in surface motility between morphs. Strains used in these experiments were alternate and original morph isolates from community A2 or F2 pass 6 (n=3 isolates per morph, all in technical triplicate; all data points shown). (A) Logistic growth rates and (D) carrying capacities were inferred from spline and logistic model fits to OD600 data respectively when isolates were grown for 24 hours in liquid NGM with 0.2X, 1X or 4X peptone. Tests are Wilcoxon comparisons of parameter estimates for alternate vs original morphs. (B) Logistic growth rates and (E) carrying capacity vs maximum diameter (48h growth) on 0.35% NGM plates at each concentration of peptone. For alternate morphs, max diameter and growth rate are positively correlated (A2a6: linear slope = 4.9, p=0.017; F2a6: linear slope = 3.7, p=8.8e-06); for original morphs, there is no linear correlation (p=0.3-0.5). For alternate morphs, max diameter and saturation OD are positively correlated (A2a6: linear slope 1.21, p=0.03; F2a6: linear slope = 0.71, p=4.11e-06); for original morphs, there is no linear correlation (A2o6, p=0.17; F2o6, p=0.8). (C) Logistic growth rate and (F) carrying capacity vs maximum surface spreading rate on 0.35% NGM agar at each concentration of peptone. Maximum spreading rate and growth rate are not linearly correlated for either morph (p=0.2-0.6), nor are maximum spreading rate and carrying capacity (all p>0.2).
